# Streptavidin-Hosted Organocatalytic Aldol Addition

**DOI:** 10.3390/molecules25102457

**Published:** 2020-05-25

**Authors:** Nicolò Santi, Louis C. Morrill, Louis Y. P. Luk

**Affiliations:** 1 School of Chemistry, Main Building, Cardiff University, Cardiff CF10 3AT, UK; SantiN@cardiff.ac.uk (N.S.); MorrillLC@cardiff.ac.uk (L.C.M.); 2 Cardiff Catalysis Institute, School of Chemistry, Main Building, Cardiff University, Cardiff CF10 3AT, UK

**Keywords:** organocatalysis, streptavidin, artificial enzyme, protein, aldol, enamine, catalysis, biocompatible

## Abstract

In this report, the streptavidin-biotin technology was applied to enable organocatalytic aldol addition. By attaching pyrrolidine to the valeric motif of biotin and introducing it to streptavidin (Sav), a protein-based organocatalytic system was created, and the aldol addition of acetone with *p*-nitrobenzaldehyde was tested. The conversion of substrate to product can be as high as 93%. Although the observed enantioselectivity was only moderate (33:67 er), further protein engineering efforts can be included to improve the selectivity. These results have proven the concept that Sav can be used to host stereoselective aldol addition.

## 1. Introduction

Representing a major mode of carbon–carbon bond formation, aldol addition is an appealing tool that has been broadly used in chemical and synthetic biology research [1,2,3]. Important applications include chemo-enzymatic synthesis of chiral synthons and bioactive chemicals [4,5,6,7,8,9,10], as well as labelling of biomolecules (protein, DNA and RNA) [3,11]. Given its usefulness, different approaches have been developed to mediate aldol addition [4,12,13,14,15,16]. Naturally occurring and de novo aldolases are excellent options. Most of these enzymes contain a catalytic active lysine residue that forms an enamine intermediate as nucleophile for conjugation [17,18]. Though often recognized for their catalytic efficiency, significant engineering efforts are typically needed in order to accept relevant substrates. Hence, the use of alternative catalysts has been examined.

Many secondary amine organocatalysts are known for their abilities to perform aldol addition [19,20,21]. Since they tend to accept a broader range of substrates and have stronger nucleophilicity than primary amines [22], it is of great interest to enable secondary amine organocatalytic aldol addition in biological contexts [23]. One simple solution is to incorporate catalytic amines within a recombinant protein as a means to enhance the biocompatibility of organocatalysis [17,24,25,26,27]. Indeed, the *N*-terminal proline of 4-oxalocrotonate tautomerase (4-OT) and its variants have been used as protein-based organocatalytic systems for stereoselective aldol addition [12,15]. Though efficient, the 4-OT system is largely limited to the use of *N*-terminal proline. However, as illustrated by traditional organocatalysis studies, modifications of the catalyst motif can lead to significant improvement in performance (selectivity and reactivity) [28,29,30,31,32,33,34,35,36]. To this end, it is of fundamental interest to explore other protein-based systems, where secondary amine catalysts other than *N*-terminal proline can be used.

Streptavidin (Sav) is an ideal host for chemical catalysis [23,24,37,38,39,40,41]. As the valeric acid motif of biotin is largely not involved in binding, it can be covalently added with an organocatalyst and introduced to Sav. Consequently, streptavidin can be used to host chemical catalysis [23,24,37,42], and applications ranging from dynamic kinetic resolution and biomolecular labeling to molecular switch design can be established [43,44,45,46,47,48]. Importantly, it has been demonstrated that nucleophiles can be generated via organocatalysis within Sav [24]. Indeed, biotin can be modified with pyrrolidine and introduced into Sav, creating a protein-based secondary amine organocatalytic system [24]. While this catalytic motif (catalyst **1**) contains essentially no bulky or H-bonding substituents adjacent to the reacting nitrogen atom, this Sav-based system was able to mediate carbon–carbon bond formation (i.e., 1,4-Michael addition) in good yield and stereoselectivity [24]. Bringing these findings together, Sav is seemingly an ideal host for organocatalytic aldol addition reactions. 

**Figure 1:**
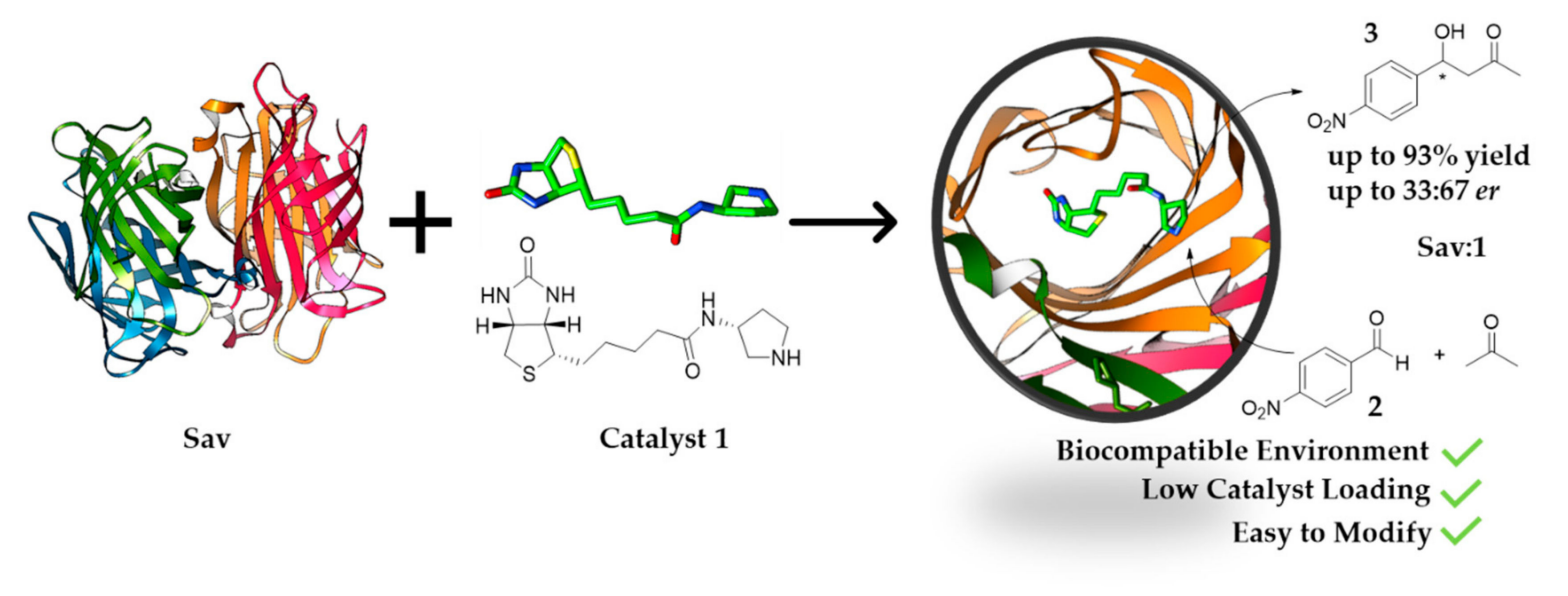
Streptavidin (Sav):**1** assembly (1 mol%, (PDB: 6GH7, 1.08 Å)) used for aldol addition reaction under biocompatible conditions.

Here, we developed an alternative Sav-based system for aldol addition. By using biotinylated pyrrolidine, Sav is converted into a system suitable for the organocatalytic aldol addition reaction between acetone and *p*-nitrobenzaldehyde derivatives (Figure 1). Using only 1 mol% of catalyst, the estimated conversion can reach up to 93%. This work demonstrates that aldol addition can be achieved using the streptavidin-biotin technology.

## 2. Results and Discussion

### 2.1. Screening for Optimised Conditions

**Scheme 1:**
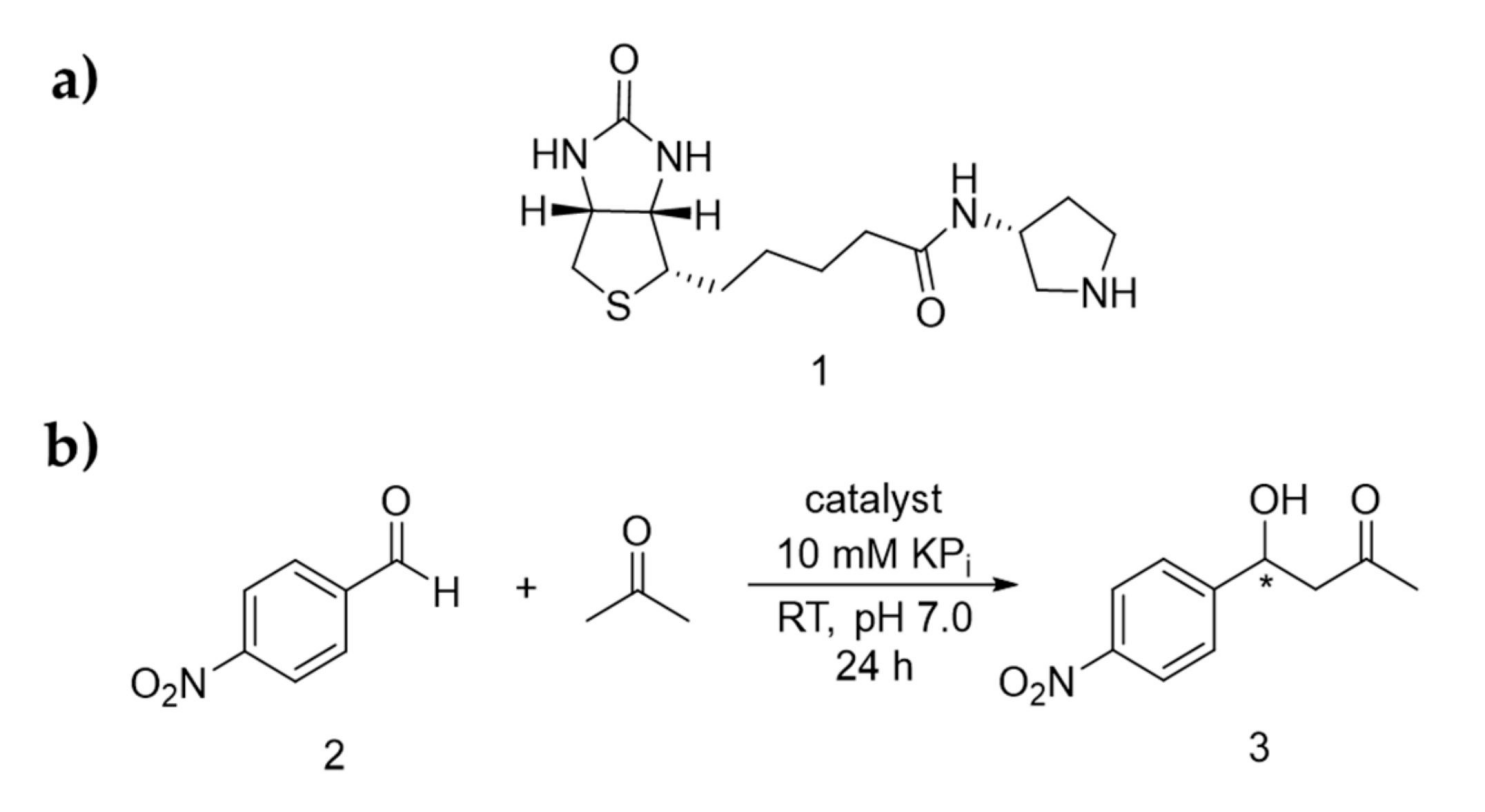
(**a**) Structure of the biotinylated catalyst **1**. (**b**) Enamine catalysed aldol addition reaction presented in this report.

Previously, a family of biotinylated secondary amine catalysts were prepared, including ones that contain imidazolidinone, proline and pyrrolidines [24]. We learned that all the biotinylated imidazolidinones were relatively difficult to prepare, involving multiple chemical steps and chromatographic steps. Furthermore, they are not active in different reactions, including hydride transfer, aromatic substitution, and Michael addition (data not shown here). Similarly, all the proline derivatives, though simpler to prepare, are not active under neutral pH. Hence, in this work, ligand **1**, which bears a minimally substituted pyrrolidine moiety, was tested for aldol addition (Scheme 1a). *p*-Nitrobenzaldehyde (**2**) was initially used as it contains a reactive carbonyl group and do not possess any enolisable α-hydrogen, whereas acetone was chosen as the nucleophile for C-C bond formation (Scheme 1b). 

In the absence of catalyst or protein, 17% racemic product (**3**) was observed when one equivalent of **2** was added to buffer (KP_i_ 10 mM at pH 7.0) containing 20 vol% of acetone (>400 equivalent), as accessed by ^1^H-NMR spectroscopy (Table 1, Entry 1). When the biotinylated organocatalyst **1** was included at 1 mol%, the reaction yield increased to 36% (Entry 2). Interestingly, when Sav but not the catalyst was included, the reaction yield was also mildly enhanced from 16% to 33% with increasing protein loading (0.1 mol%–1 mol%, Entry 3–5). In any case, no enantioselectivity was observed.

To see if the aldol addition reaction could proceed organocatalytically, both streptavidin and ligand **1** (1:1 ratio) were included and the reaction yield increased up to 93%. Furthermore, the enantiomeric ratio (er) of *R* to *S* isomer was measured to be 33:67, thus suggesting that stereoselectivity of the aldol addition was originated from the binding of ligand **1** to Sav (Entry 6–8). There was also a small amount of elimination product observed (5%, see Section 6.1.8 in the SI). Similar to previous observation [24], enantioselectivity was only observed when Sav was included, thereby providing evidence that the organocatalytic reaction takes place within the protein scaffold. With the goal to further enhance stereoselectivity, we modified the reaction conditions, including the addition of co-catalyst trifluoroacetic acid (1%, [49,50,51] Entry 9) and decrease of the reaction temperature (10 °C, Entry 10). Changing the amount of acetone used or including the amount of cosolvents (i.e., methanol, acetonitrile, and isopropanol) did not lead to improved stereoselectivity (Entry 11–16). Nevertheless, in all cases, neither the yield nor selectivity was improved.

### 2.2. Site-Directed Mutagenesis of the Protein Host

Based on previous analysis via X-ray crystallography and molecular dynamics (MD) studies [24], Ser112 and Lys121 were found to be in proximity to the pyrrolidine catalyst, and these residues were modified to see if performance of the aldol addition reaction can be affected (Table 2). 

A reduced tetrameric streptavidin variant (T-rSav) was used to create the variants [47]. Ser112 was found to be in hydrogen-bonding distance with the pyrrolidine motif [24]. When this residue was modified to glutamate, a vastly different electrostatically charged residue, the resulting S112E variant, was found to be a poor host, producing a poor reaction yield (14%). On the other hand, Lys121 was found to be in close proximity to the tetrahedral intermediate [24]. When this residue was replaced with a non-charged and small residue such as alanine, the resulting protein-based complex could yield 89% of the aldol product. However, poor stereoselectivity was observed.

## 3. Materials and Methods

Reactions were performed in oven dried glassware without precautions to exclude air. Reaction temperatures are stated as heating device temperature (e.g., oil bath, shaker, etc.), if not stated otherwise. Concentrations under reduced pressure were performed by rotary evaporation at 40 °C at the appropriated pressure, unless otherwise noted. Deionized water was obtained by a PURELAB^®^ Option system (15 MΩ cm, ELGA LabWater, High Wycombe, UK). Analytical and preparative thin layer chromatography (TLC) was carried out with silica gel 60 F254 aluminum sheets (Merck KGaA, Darmstadt, Germany). Detection was carried out using UV light (λ = 254 nm and 366 nm), followed by immersion in permanganate staining solution with subsequent development via careful heating with a heat gun. Flash column chromatography was performed using silica gel (pore size 60 Å, 0.040–0.063 mm, Global Life Sciences Solutions USA LLC, Marlborough, USA).

*para*-Nitrobenzaldehyde (**2**) for aldol addition reactions with Sav was obtained commercially (Merck KGaA, Darmstadt, Germany) and if necessary purified by washes with sodium bicarbonate pH 8.3, subsequent drying with magnesium sulphate and stored under inert atmosphere at 4 °C. All other solvents and reagents were obtained from commercial sources and used as received.

Sav (Streptavidin *Streptomyces avidinii* recombinant, tetramer, *M*_w_ ≈ 52 kDa), a recombinant variant of streptavidin processed at the C and N termini and carrying amino acids 13–139, was obtained commercially (PRO-791, ProSpec-Tany TechnoGene Ltd., Ness-Ziona, Israel) as lyophilized powder in 10 mm KP_i_ pH 6.5 and stored at −23 °C upon receipt until further use. According to the supplier Sav has the following amino acid sequence: 

MAEAGITGTWYNQLGSTFIVTAGADGALTGTYESAVGNAESRYVLTGRYDSAPATDGSGTALGWTVAWKNNYRNAHSATTWSGQYVGGAEARINTQWLLTSGTTEANAWKSTLVGHDTFTKVKPSAAS

The plasmid for tetrameric reduced streptavidin (T-rSav) (encoding for a Streptavidin *Streptomyces avidinii* recombinant, tetramer, *M*_w_ ≈ 50 kDa, “reduced” Streptavidin with amino acids 16–133) was obtained as gift from Takeshi Sano (pTSA-13, Addgene plasmid #17327, http://n2t.net/addgene:17327, RRID:Addgene_17327) [47]. The gene encoding for *T-rSav* translates to the following amino acid sequence: 

MGITGTWYNQLGSTFIVTAGADGALTGTYESAVGNAESRYVLTGRYDSAPATDGSGTALGWTVAWKNNYRNAHSATTWSGQYVGGAEARINTQWLLTSGTTEANAWKSTLVGHDTFTKV

A 3510 benchtop pH Meter (VWR International, Radnor, USA) connected to a Universal pH electrode (VWR International, Radnor, USA) was used for the pH adjustment of buffers and reaction mixtures employing either 1.0 M or 0.1 M sodium hydroxide solution or hydrochloric acid. Shaking of the reactions (300 rpm) at 25 °C was achieved using a thermoshaker Mini shake lite (VWR International, Radnor, USA) or a Incubating Orbital Shaker (VWR International, Radnor, USA). ^1^H- and ^13^C-NMR spectra were recorded in CDCl_3_ or DMSO-*d*_6_ on Bruker Fourier 300, Ultrashield 400, or Ascend 500 instruments (Bruker Corporation, Billerica, USA). Chemical shifts are reported in parts per million (ppm) and are referenced to the residual solvent resonance as the internal standard (CHCl_3_: δ = 7.26 ppm for ^1^H; DMSO: δ = 2.54 ppm for ^1^H). Data are reported as follows: chemical shift (δ), multiplicity (br s = broad singlet, s = singlet, d = doublet, dd = double doublet, td = triple doublet, t = triplet, dt = double triplet, q = quartet, p = pentet, sept = septet, br m = broad multiplet, m = multiplet, mc = centrosymmetric multiplet), coupling constants (Hz) and integration. Size exclusion chromatography was performed using a ÄKTA Purifier workstation (Global Life Sciences Solutions USA LLC, Marlborough, USA) system with the respective column mentioned in the detailed procedure.

### 3.1. Experimental Details for the Synthesis of Catalyst ***1***

**Scheme 2:**
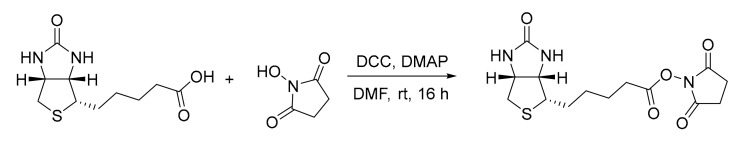
Synthesis of (+)-biotin NHS ester by coupling reaction.

**Scheme 3:**
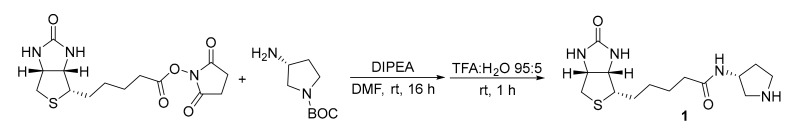
Synthesis of catalyst **1**.

Catalyst **1** was synthesized following a two-step procedure previously reported from our group (Scheme 2 and Scheme 3) [24].

Synthesis and ^1^H-NMR Assignment for the Synthesis of (+)-Biotin NHS ester

(+)-Biotin (960 mg, 4.0 mmol, 1.0 eq), *N*-hydroxysuccinimide (NHS, 920 mg, 8.0 mmol, 2.0 eq), and 2-(dimethylamino)pyridine (DMAP, 24 mg, 0.2 mmol, 0.05 eq) were dissolved in dry DMF (ca. 40 mL) under inert atmosphere. The solution was cooled to 0 °C with an ice bath. Dicyclohexylcarbodiimide (DCC, 908 mg, 4.4 mmol, 1.1 eq) dissolved in dry DMF was then added dropwise. The reaction mixture was left stirring at room temperature overnight and formed a precipitate was removed via vacuum filtration. Crude NHS ester was precipitated by the addition of Et_2_O, collected by filtration, and washed with deionized water and Et_2_O. The crude product was recrystallized from iso-propanol, collected via filtration, and washed with Et_2_O to afford the active ester as white solid after drying under fine vacuum (272 mg, 0.8 mmol, 20% yield).

^1^H-NMR (400 MHz, DMSO-d6): δ = 6.43 (s, 1H), 6.37 (s, 1H), 4.32 (mc, 1H), 4.13 (mc, 1H), 3.10 (mc, 1H), 2.87–2.78 (m, 5H), 2.67 (t, *J* = 7.3 Hz, 1H), 2.58 (d, *J* = 12.6 Hz, 1H), 1.71–1.28 (m, 6H) ppm. The analytical data are in accordance with the literature [24].

Synthesis and ^1^H-NMR Assignment for the Synthesis of (*R*)-3-(5-((3a*S*,4*S*,6a*R*)-2-oxohexahydro-1H-thieno[3,4-d]imidazol-4-yl)pentanamido)pyrrolidin-1-ium formate (Catalyst **1**)

(+)-Biotin NHS ester (100 mg, 0.29 mmol, 1.0 eq) and (3*R*)-3-amino-1-Boc-pyrrolidine (64 mg, 0.29 mmol, 1.0 eq) were dissolved in dry DMF. Di-iso-propylethylamine (DIPEA, 106 µL, 0.58 mmol, 2.0 eq) was added and the reaction was allowed to proceed under stirring overnight at room temperature. The solvent was removed under reduced pressure at 80 °C. The residue was dissolved in a mixture of trifluoroacetic acid (TFA):H_2_O (95:5, 5.0 mL) and stirred at room temperature for 1 h. Excess TFA was removed under reduced pressure, the residue was dissolved in a minimal amount of H_2_O and lyophilized. The crude product was purified by preparative HPLC (High Performance Liquid Chromatography) using a Supelcosil C18 column (25 cm × 21.2 mm, 12 µm; gradient H_2_O:MeCN 99:1 to 50:50 over 30 min, 0.1% HCO_2_H, 10 mL·min^−1^, 20 °C, λ = 210 nm, Merck KGaA, Darmstadt, Germany) and catalyst **1** was obtained as white solid (43 mg, 0.12 mmol, 42% yield).

^1^H-NMR (400 MHz, D_2_O): δ = 4.57 (dd, *J* = 7.8, 4.9 Hz, 1H), 4.45–4.35 (m, 2H), 3.54 (dd, *J* = 12.5, 7.0 Hz, 1H), 3.46–3.35 (m, 2H), 3.30 (dt, *J* = 9.6, 4.1 Hz, 1H), 3.19 (dd, *J* = 12.5, 4.9 Hz, 1H), 2.95 (dd, *J* = 13.1, 4.9 Hz, 1H), 2.74 (d, *J* = 13.1 Hz, 1H), 2.38–2.27 (m, 1H), 2.23 (t, *J* = 7.2 Hz, 2H), 1.99 (td, *J* = 13.5, 6.8 Hz, 1H), 1.76–1.47 (m, 4H), 1.45–1.31 (m, 2H) ppm. The analytical data are in accordance with the literature [24].

### 3.2. Experimental Details for the Preparation and Purification of T-rSav and Mutants

Tetrameric reduced streptavidin (T-rSav) and relative mutants were expressed using an *E. coli* expression system with the following protocol. Plasmid pTSA-13 containing the desired *T-rSav* gene in a pET-3a vector was transformed into calcium competent BL21(DE3) pLysS cells and grown for 16 h on LB agar plates containing 100 μg/mL ampicillin and 34 μg/mL chloramphenicol. A single colony from the plate was picked to inoculate a 15 mL MTP (per 1 L: 10 g tryptone, 10 g NaCl, 5 g yeast extract, 2.2 g Na_2_HPO_4_, 1 g KH_2_PO_4_, pH = 6.9) starter culture containing 100 μg/mL ampicillin and 34 μg/mL chloramphenicol, which was incubated at 37 °C and 180 rpm overnight. The culture was diluted to 40 mL with 20% glucose and then added to 1 L MTP medium containing 100 μg/mL ampicillin and 34 μg/mL chloramphenicol, yielding a final glucose concentration of 0.05%. The cultures were grown at 37 °C and 225 rpm to an OD_600_ of 1.0–1.2 and induced with IPTG (Isopropyl β-D-1-thiogalactopyranoside) at a final concentration of 1 mM. The culture was grown at 25 °C for 16 h and the cell pellet was harvested after centrifugation at 4000 rpm at 4 °C for 25 min and stored at −20 °C.

The pellet was subjected to a freeze–thaw cycle, resuspended in 25 mL of lysis buffer (50 mM Tris, 100 mM NaCl, 1 mM PMSF, pH 8.0) and lysed by sonication (7 min, 5 s on, 10 s off). The insoluble fraction was isolated by centrifugation at 15000 rpm for 25 min at 4 °C. The supernatant was discarded and the insoluble fraction was washed with wash buffer 1 (4× resuspension in 50 mM Tris, 110 mM EDTA, 1.5 M NaCl, 1 mM PMSF, 0.1% Triton X-100, pH 8.0 and pellet re-isolation by centrifugation at 11000 rpm and 4 °C) and wash buffer 2 (4× resuspension in 50 mM Tris, 110 mM EDTA, 1.5 M NaCl, 1 mM PMSF, pH 8.0 and pellet re-isolation by centrifugation at 11000 rpm and 4 °C). The insoluble fraction was resuspended in denaturing buffer 1 (5 mL/g pellet, 6 M GdnHCl, 50 mM Tris-HCl, pH 1.5) and incubated at 37 °C and 180 rpm for 16 h. The insoluble fraction was removed by centrifugation at 15000 rpm and 4 °C. The supernatant was diluted to 200 mL with denaturing buffer 2 (6 M GdnHCl, 50 mM Tris-HCl, pH 6.5) and dialysed against 3 L of 6 M GdnHCl, 50 mM Tris-HCl, pH 6.5 for 3 h at room temperature. The dialysis bag was then placed into fresh 3 M GdnHCl, 50 mM Tris-HCl, pH 6.5 (denaturing buffer 2 was reused up to 5 times). T-rSav was refolded by gradient dialysis, pumping in refolding buffer (0.5 mg/L catalyst **1**, 10 mM KP_i_, pH 7.0) at 4 mL/min, constant stirring of the mixture at 4 mL/min for 48 h at room temperature. Towards the end of this process a varying amount of precipitation was observed. The precipitate was removed via centrifugation at 15000 rpm at 4 °C and the supernatant was concentrated to 20 mL by Amicon ultra centrifugation using a 3.5 kDa cut-off. The concentrated solution was transferred into a centrifugal concentrator with a 10 kDa cut-off and the buffer was exchanged five times by concentration to 2.5 mL and refilling to 20 mL (10 mM KP_i_, pH 7.0). The protein solution was finally concentrated to obtain a protein concentration of 2 mg/mL as determined by nanodrop (Thermo Fischer Scientific, Waltham, USA) measurement at 210 nm. This was used for catalysis of the aldol addition reaction without further purification. A sample of the solution was loaded on SDS-PAGE to check the purity (>95%, see pg S2, Figure S1) of the protein (15% *w*/*v*).

The mutations K121 or S112 were introduced by site-directed mutagenesis PCR using PrimeStar HS DNA polymerase (Takara Bio Inc., Kusatsu, Japan) and the accompanying buffers, dNTPs and primers mentioned in Table 3 below. Due to the high GC content of the region of interest a variety of methods and temperatures had to be screened, as primer insertions were observed, especially for mutations at K121. Hence, a 50 µL PCR was prepared according to the instructions and the reaction mixture distributed equally (12.5 µL) over 4 PCR tubes. These were then subjected to the following conditions, using a gradient to achieve a different annealing temperature for each tube. Method 1: Initial denaturing (4 min, 95 °C), 33 cycles of (10 s at 98 °C, 5 s at 58/60/62/64 °C, 5 min at 72 °C), final extension (10 min, 72 °C) and hold (4 °C). Method 2: Initial denaturing (4 min, 95 °C), 15 cycles of (10 s at 98 °C, 5 s at 58/60/62/64 °C, 5 min at 72 °C), 15 cycles of (10 s at 98 °C, 5 s at 61/63/65/67 °C, 5 min at 72 °C) and final extension (5 min, 72 °C) and hold (4 °C). Method 3: Initial denaturing (4 min, 95 °C), 3 cycles of (10 s at 98 °C, 5 s at 55/57/59/61 °C, 5 min at 72 °C), 3 cycles of (10 s at 98 °C, 5 s at 58/60/62/64 °C, 5 min at 72 °C), 30 cycles of (10 s at 98 °C, 5 s at 61/63/65/67 °C, 5 min at 72 °C) and final extension (10 min, 72 °C) and hold (4 °C). In the case of the K121 mutation, Method 1 and 3 were also applied using 3% DMSO (Dimethyl sulfoxide), if no positive results were obtained without DMSO. The mutant constructs were confirmed by DNA sequencing (Eurofins Genomics Germany GmbH, Ebersberg, Germany) using the T7 promoter primer (TAATACGACTCACTATAGG).

### 3.3. Experimental Details for the Activity Screening of Catalysts 1 for the Aldol Addition Reaction of Acetone and p-Nitrobenzaldehyde

**Scheme 4:**
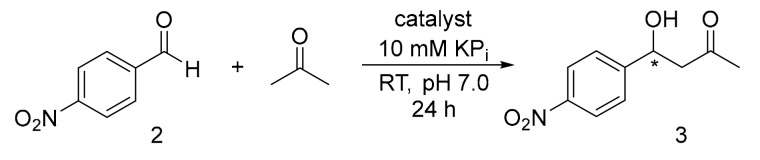
Aldol addition reaction of acetone to *p*-nitrobenzaldehyde.

^1^H-NMR Based Screening for Yield Determination of Aldol Addition Reaction of Acetone and *p*-Nitro benzaldehyde (Scheme 4).

A stock solution of catalyst **1** (0.50 mg, 1.59 µmol) was prepared dissolving the catalyst in 1 mL of KPi (pH 7.0, 10 mM) into a 1.5 mL Eppendorf tube. *p*-Nitrobenzaldehyde (**2**, 20 mg, 132.34 µmol) was dissolved in 1 mL of acetone to create a stock solution. Commercial Sav (0.58 mg, 1 mol%, 33 nmol) was weighted into a 1.5 mL Eppendorf tube and dissolved in 379.25 µL of KP_i_ (pH 7.0, 10 mM). An aliquot of 20.75 µL (1 mol%) of the catalyst stock solution was added to the Sav Eppendorf tube. Subsequently, an aliquot of 24.93 µL (1 eq.) of the *p*-nitrobenzaldehyde stock solution was added to the Eppendorf tube. An amount of 75.07 µL of acetone was added to reach a final acetone volume of 100 µL. The mixture was shaken at 300 rpm at 25 °C for 24 h. The mixture was extracted with CH_2_Cl_2_ (500 × 3 µL) and the organic phase evaporated under reduced pressure. The crude of reaction was dissolved in CDCl_3_ (620 µL) and subjected to ^1^H-NMR analysis.

### 3.4. Synthesis and ^1^H-NMR Assignment of Aldol Addition Reaction Product

**Scheme 5:**
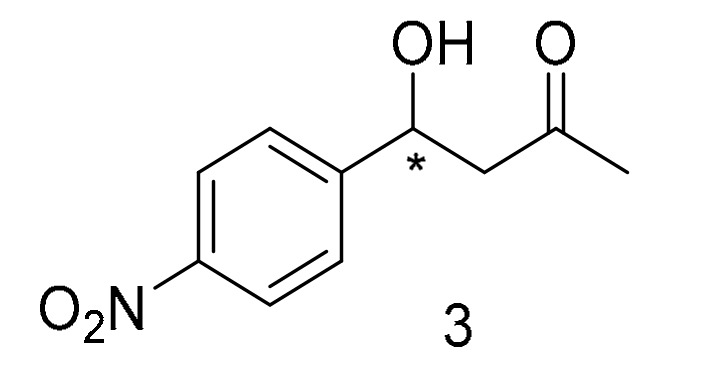
4-hydroxy-4-(4-nitrophenyl) butan-2-one.

Synthesis and ^1^H-NMR assignment of 4-hydroxy-4-(4-nitrophenyl) butan-2-one (**3**) (Scheme 5).

4-hydroxy-4-(4-nitrophenyl) butan-2-one (**3**) was synthesised as previously reported (see pg S3, Figure S2) [48]. ^1^H-NMR (400 MHz, CDCl_3_): δ = 8.20 (d, *J* = 8.8 Hz, ArH, 2H), 7.53 (d, *J* = 8.8 Hz, ArH, 2H), 5.26 (m, ArCH(OH)CH_2_, 1H), 3.61 (br s, ArCH(OH)CH_2_, 1H), 2.85 (m, CH_2_COCH_3_, *2*H), 2.22 (s, CH_2_COCH_3_, *3*H) ppm. The analytical data were found to be in good agreement with the reported data [48].

### 3.5. Chiral HPLC Data of Activity and Selectivity Screening

#### Screening Reactions

Analytical chiral HPLC analysis of product **3** was performed on a 1260 Infinity Quaternary LC system (Agilent Technologies, Santa Clara, USA) using a Lux Amylose-1 column (Phenomenex, Torrance, USA), 4.6 mm × 250 mm (0.5 mL/min, 25 °C, *n*-hexane/iso-propanol 75:25, 50 min). Slight variations on the retention time are due to the change of the column guard throughout the measurements. The peaks were assigned using signal at 280 nm.

## 4. Conclusions

In summary, we reported the use of streptavidin as a host for organocatalytic aldol addition. The reaction yield is nearly quantitative using only 1 mol% of catalyst. When compared to other protein-based aldol addition systems, such as ones catalyzed by 4-OT [12,15], catalytic antibodies [52] and RA95.5-8F [17], both the enantioselectivity and reactivity can further be improved in the Sav-based system, and thus additional protein engineering efforts are needed. However, in contrast with the other existing system, the performance of the Sav-based catalytic system can be improved by modifying both the catalytic motif and the protein scaffold. Indeed, by site-directed mutagenesis studies, this work illustrated that Ser112 and Lys121 are crucial in dictating the reaction conversion and stereoselectivity, respectively. The roles of these residues will be further investigated with the aim to improve the performance of this Sav-hosted aldol addition reaction. Hence, we anticipate this proof-of-concept study will be converted into applications for organocatalytic aldol addition in chemical and synthetic biology research in future.

## Figures and Tables

**Table 1:** Screening for assessing the best conditions to perform enamine-catalysed aldol addition.

Entry	Catalyst	Eqv. Acetone	Co-Solvent	Loading (mol%)	Estimated Conversion ^a^/%	er (*R*:*S*)
1	None	20 vol%	NA	None	17	50:50
2	**1**			1	36	50:50
3	Sav			0.1	16	50:50
4	Sav			0.5	23	50:50
5	Sav			1	33	50:50
6	Sav:**1**			0.1	47	38:62
7	Sav:**1**			0.5	79	33:67
8	Sav:**1**			1	93	33:67
9 ^b^	Sav:**1**			1	92	33:67
10 ^c^	Sav:**1**			1	37	34:66
11	Sav:**1**	5	25% MeOH	1	20	33:67
12	Sav:**1**	5	25% ACN	1	12	33:67
13	Sav:**1**	5	25% *i*-PrOH	1	1	NA
14	Sav:**1**	10		1	5	NA
15	Sav:**1**	20		1	9	36:64
16	Sav:**1**	50		1	18	33:67

^a^ The estimated conversion was calculated by measuring the integral ratio between the substrate and product peaks from the reaction crude ^1^H-NMR. ^b^ 1 mol% trifluoroacetic acid (TFA) used as additive. ^c^ Reaction run at 10 °C.

**Table 2:** Screening for assessing the best conditions to perform enamine-catalysed aldol reactions.

Catalyst	Estimated Conversion ^a^/%	er (*R*:*S*)
Sav:**1**	93	33:67
T-rSav:**1**	92	35:65
S112E:**1**	14	39:61
K121A:**1**	89	44:56

^a^ The estimated conversion was calculated by measuring the integral ratio between the substrate and product peaks from the reaction crude ^1^H-NMR.

**Table 3:** List of primers used for the introduction of mutations in tetrameric reduced streptavidin (T-rSav) at positions S112 and K121.

Mutation	Primer (5’ to 3’)
S112E	Forward GGCTGCTGACC**GAA**GGCACCACCGAGG Reverse CCTCGGTGGTGCC**TTC**GGTCAGCAGCC
K121A	Forward ACCGAGGCCAACGCCTGG**GCG**TCCACGCTGGTCGGC Reverse GGCGTTGGCCTCGGTGGTGCCGGA

## Supplementary Materials

The following are available online. Figure S1: SDS-PAGE of T-rSav and mutants, Figure S2: ^1^H-NMR spectrum for the aldol product **3**, Figure S3–S18: Chiral-LC spectra for the aldol product **3** using different catalysts and conditions, Figure S19–S37: ^1^H-NMR spectrum for aldol reactions using different catalysts and conditions.
