# Supplementary material for: Streptavidin-Hosted Organocatalytic Aldol Addition
Source: Molecules. 2020 May 25;25(10):2457. doi: 10.3390/molecules25102457 (PMC7287710; doi:10.3390/molecules25102457)
Supplement: Supplementary file 1 [file molecules-25-02457-s001.pdf]

molecules

Supporting Information

Streptavidin-Hosted Organocatalytic Aldol Addition

Nicolò Santi [1], Louis C. Morrill [1,2] and Louis Y. P. Luk [1,2,*]

[1] School of Chemistry, Main Building, Cardiff University, Cardiff, CF10 3AT, UK; SantiN@cardiff.ac.uk (N.S.); MorrillLC@cardiff.ac.uk (L.C.M.)
[2] Cardiff Catalysis Institute, School of Chemistry, Main Building, Cardiff University, Cardiff, CF10 3AT, UK
* Correspondence: lukly@cardiff.ac.uk; Tel.: +44 (0)29 2251 0161

SDS-PAGE of T-rSav and mutants

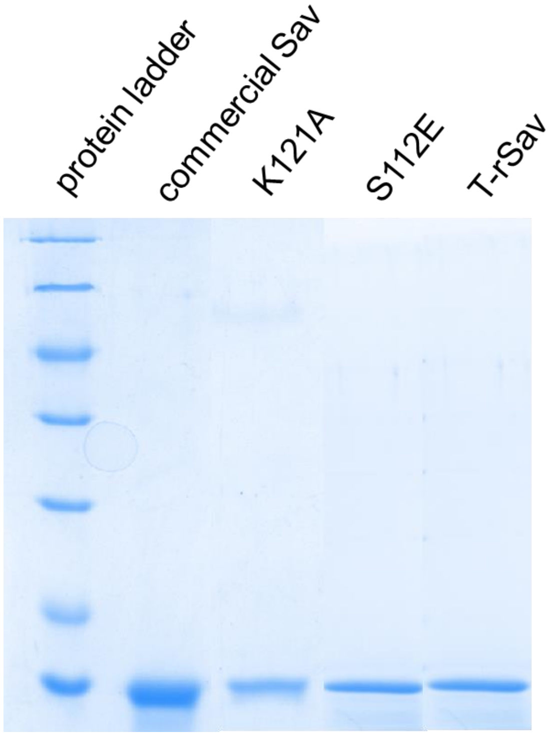

Figure S1. SDS-PAGE (15% w/v) for Sav (commercial Sav), T-rSav, K121A and S112E variants.

[1,] H NMR Spectrum of Product 3

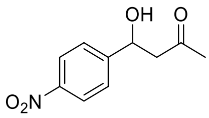

3

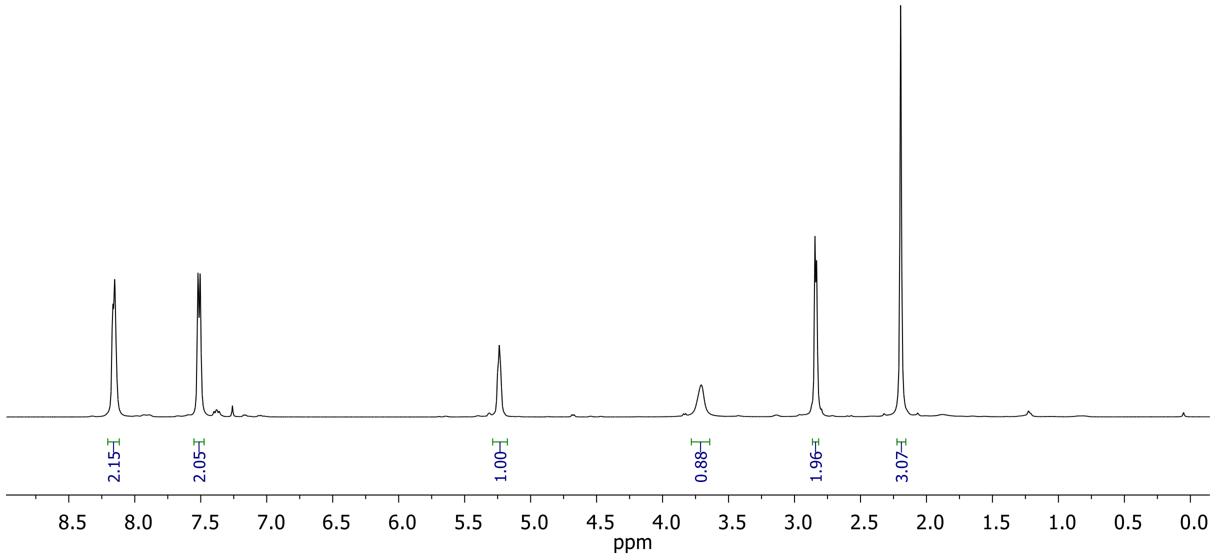

Figure S2. ^{1}H NMR spectrum for the aldol product **3**.

Chiral HPLC Data of Activity and Selectivity Screening

Screening Reactions

Racemate 3

Racemic samples of **3** were obtained following a known procedure, using piperidine as catalyst.

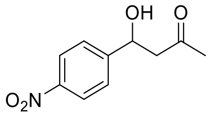

3

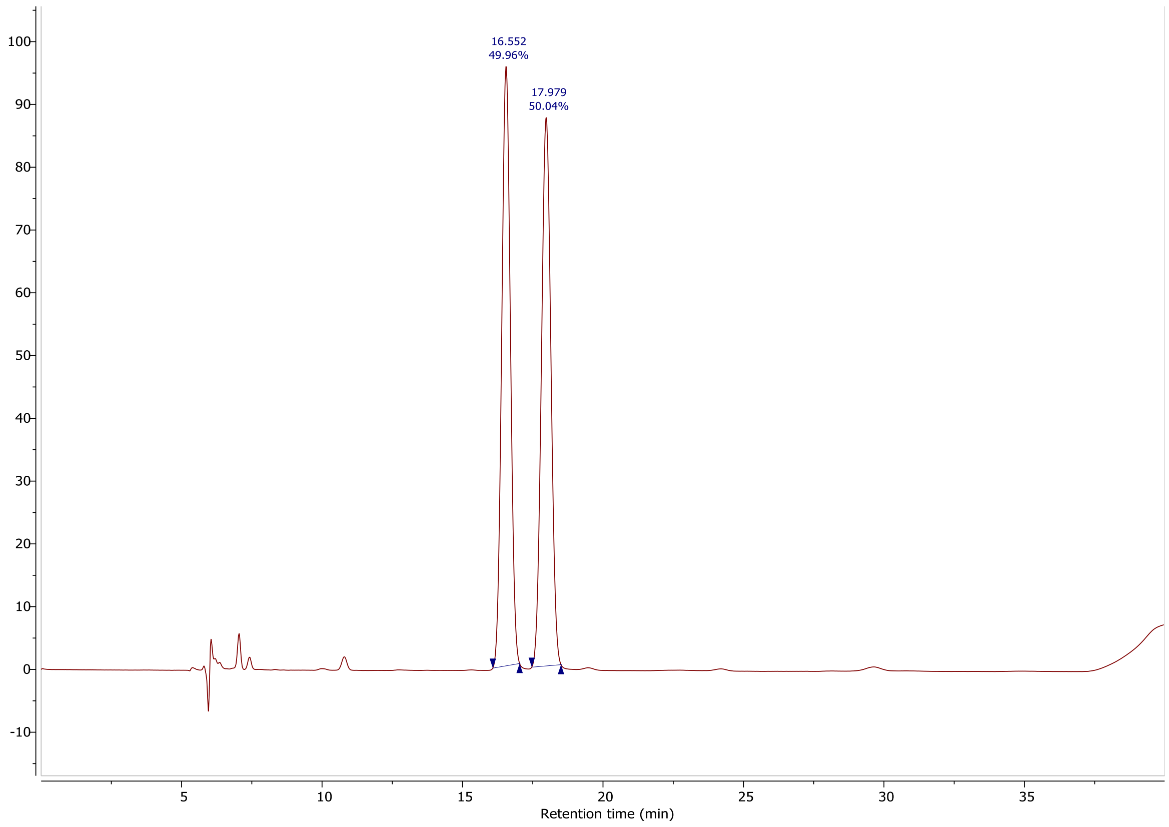

Figure S3. Chiral-LC spectrum for the racemate of product **3**

Entry	Retention time (min)	Peak Area (%)
1 (*R*-enantiomer)	16.5	50.0
2 (*S*-enantiomer)	18.0	50.0

L-proline

The absolute stereochemistry of **3** was assigned after running the sample obtained using L-Proline as catalyst.

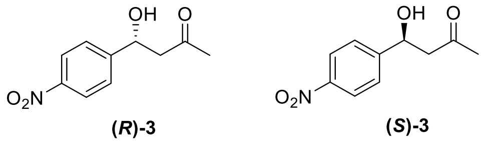

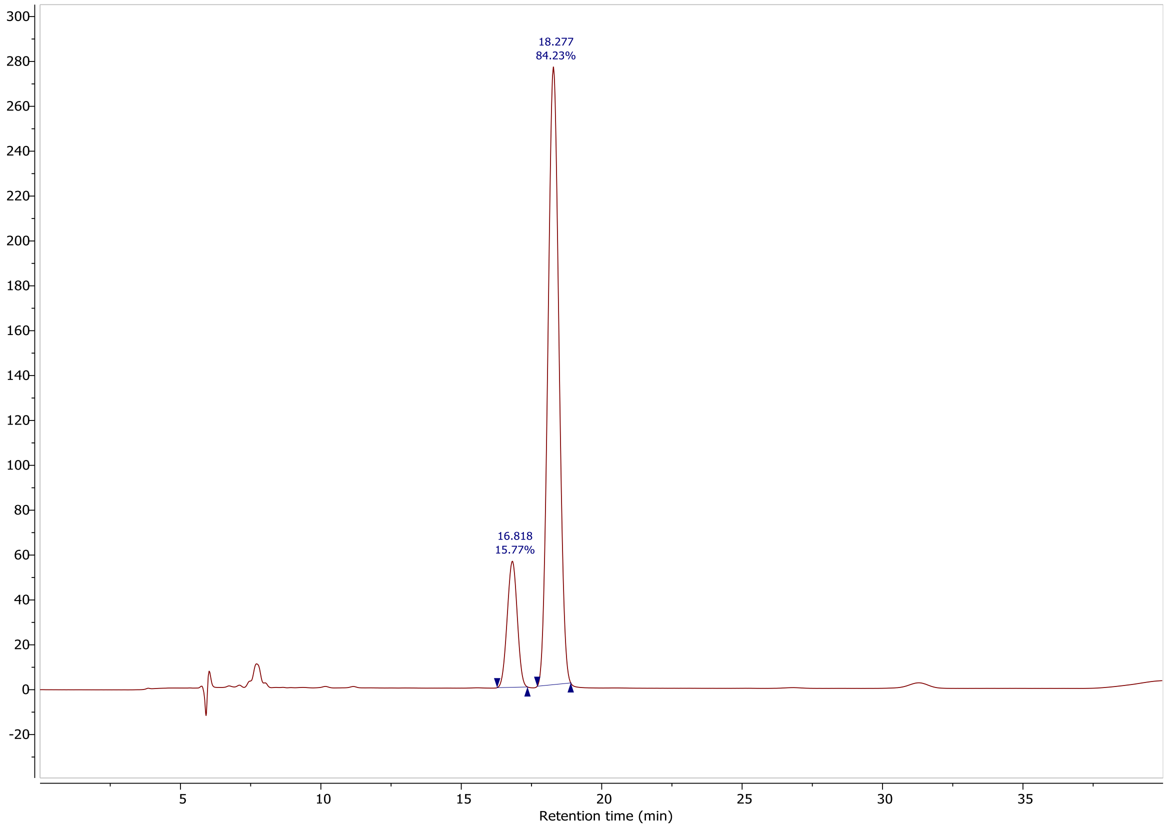

Figure S4. Chiral-LC spectrum for product **3** obtained using L-Proline.

Entry	Retention time (min)	Peak Area (%)
1 (*R*-enantiomer)	16.8	15.7
2 (*S*-enantiomer)	18.3	84.3

Catalyst 1 (1 mol%)

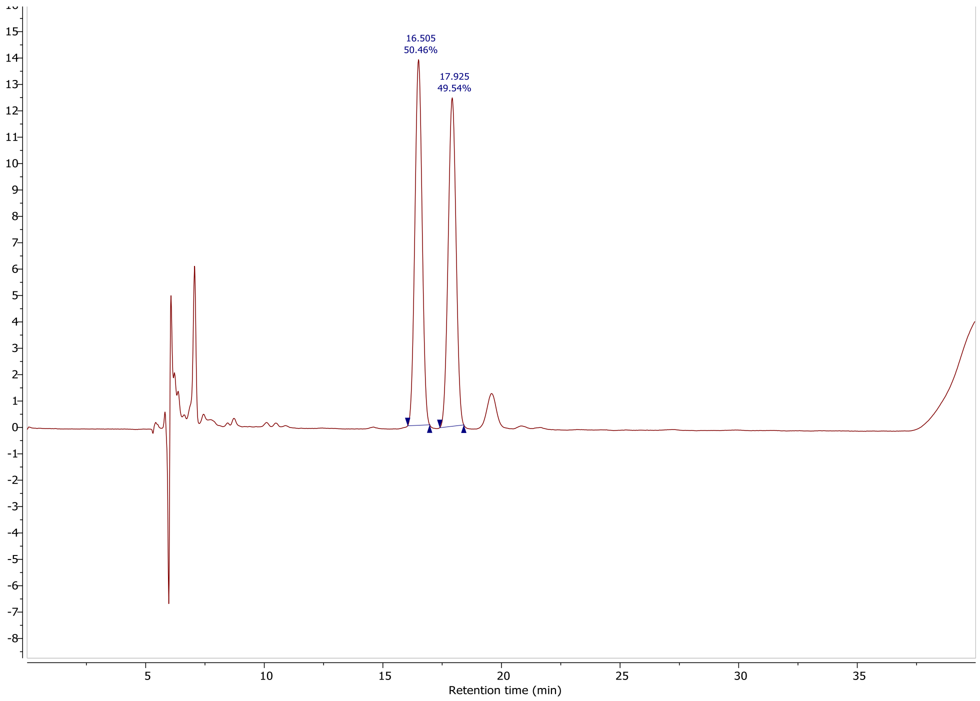

Figure S5. Chiral-LC spectrum for product **3** obtained using catalyst **1**.

Entry	Retention time (min)	Peak Area (%)
1 (*R*-enantiomer)	16.5	50.5
2 (*S*-enantiomer)	17.9	49.5

Sav (1 mol%)

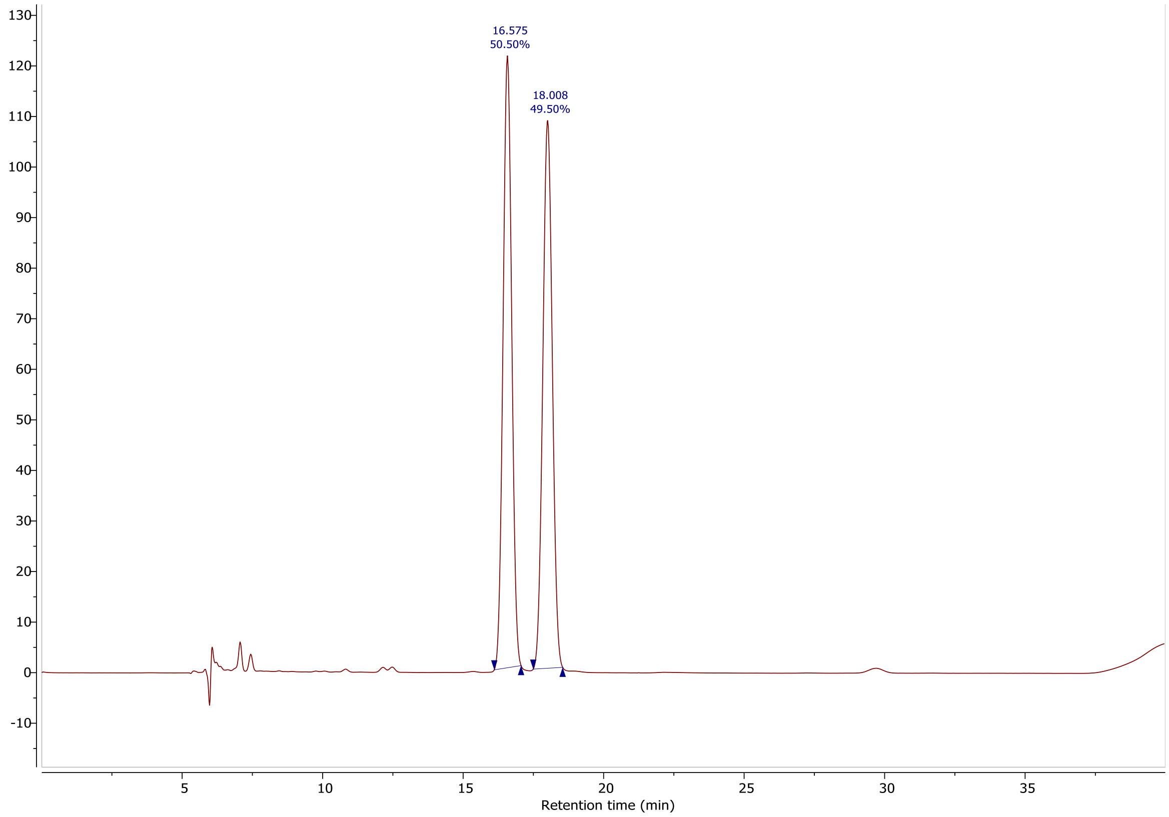

Figure S6. Chiral-LC spectrum for product **3** obtained using Sav.

Entry	Retention time (min)	Peak Area (%)
1 (*R*-enantiomer)	16.5	50.5
2 (*S*-enantiomer)	18.0	49.5

Sav:1 (0.1 mol%)

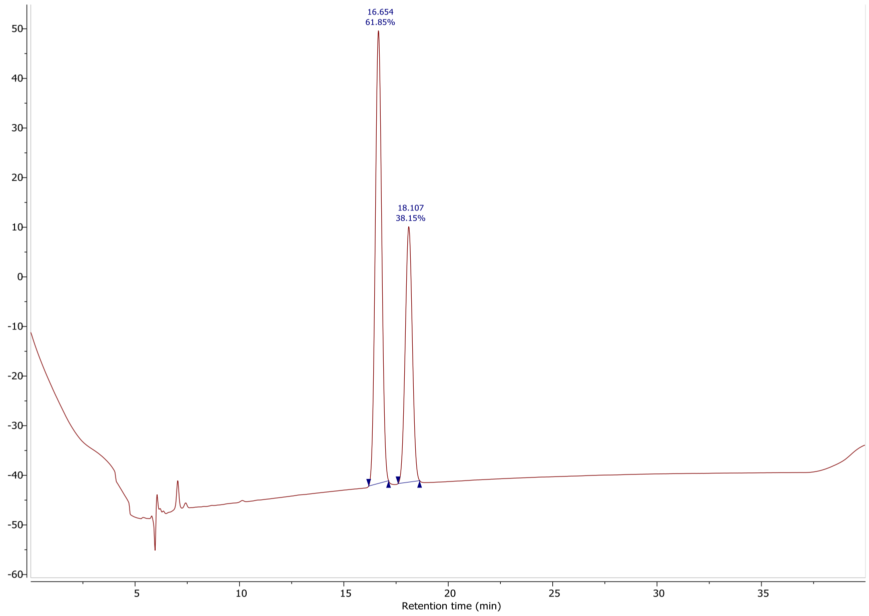

Figure S7. Chiral-LC spectrum for product **3** obtained using Sav:**1** (0.1 mol%).

Entry	Retention time (min)	Peak Area (%)
1 (*R*-enantiomer)	16.6	61.9
2 (*S*-enantiomer)	18.1	38.1

Sav:1 (0.5 mol%)

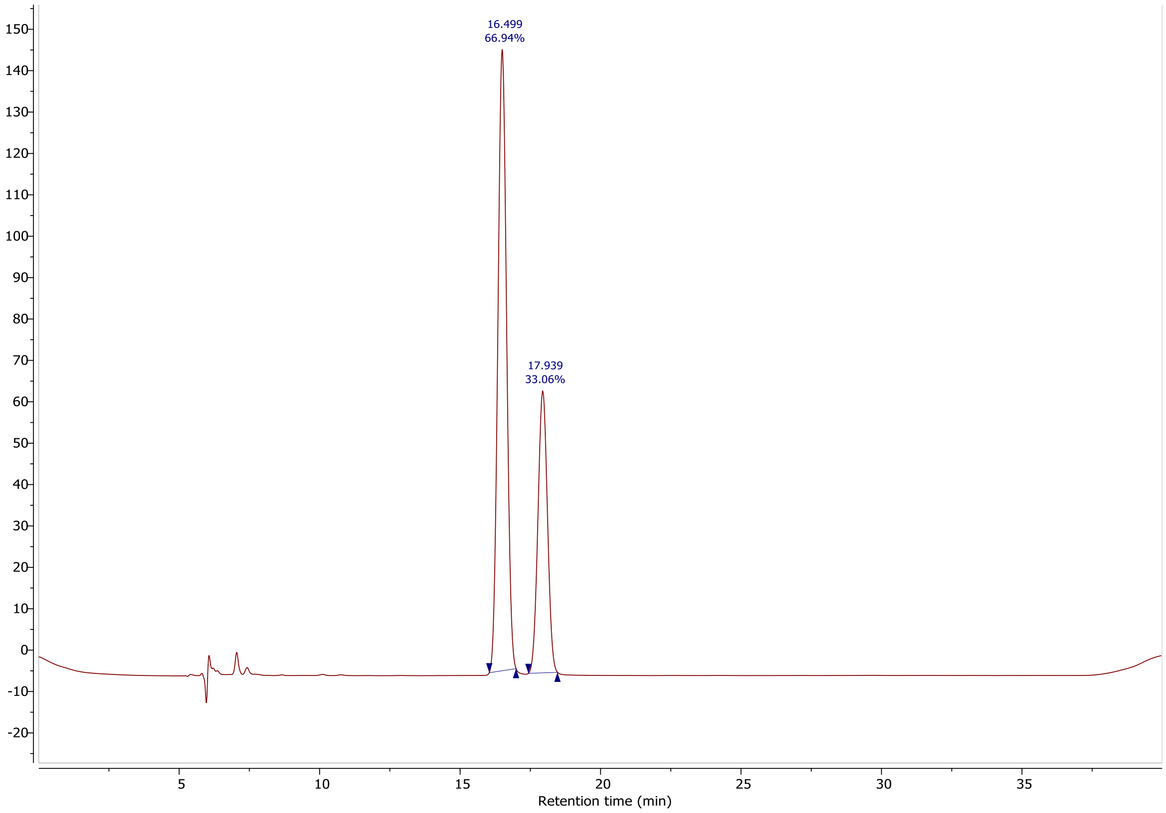

Figure S8. Chiral-LC spectrum for product **3** obtained using Sav:**1** (0.5 mol%).

Entry	Retention time (min)	Peak Area (%)
1 (*R*-enantiomer)	16.5	67.0
2 (*S*-enantiomer)	17.9	33.0

Sav:1 (1 mol%)

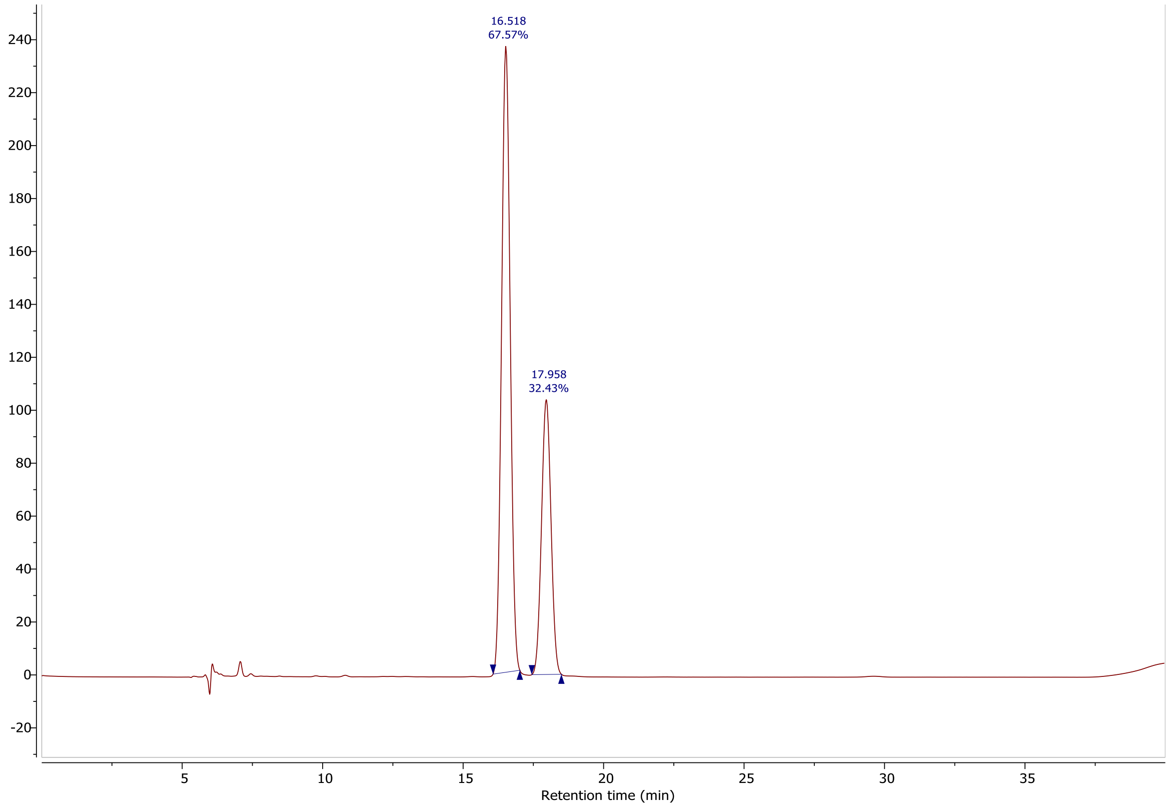

Figure S9. Chiral-LC spectrum for product **3** obtained using Sav:**1** (1 mol%).

Entry	Retention time (min)	Peak Area (%)
1 (*R*-enantiomer)	16.5	67.6
2 (*S*-enantiomer)	17.9	32.4

Sav:1 (1 mol% + 1mol% TFA)

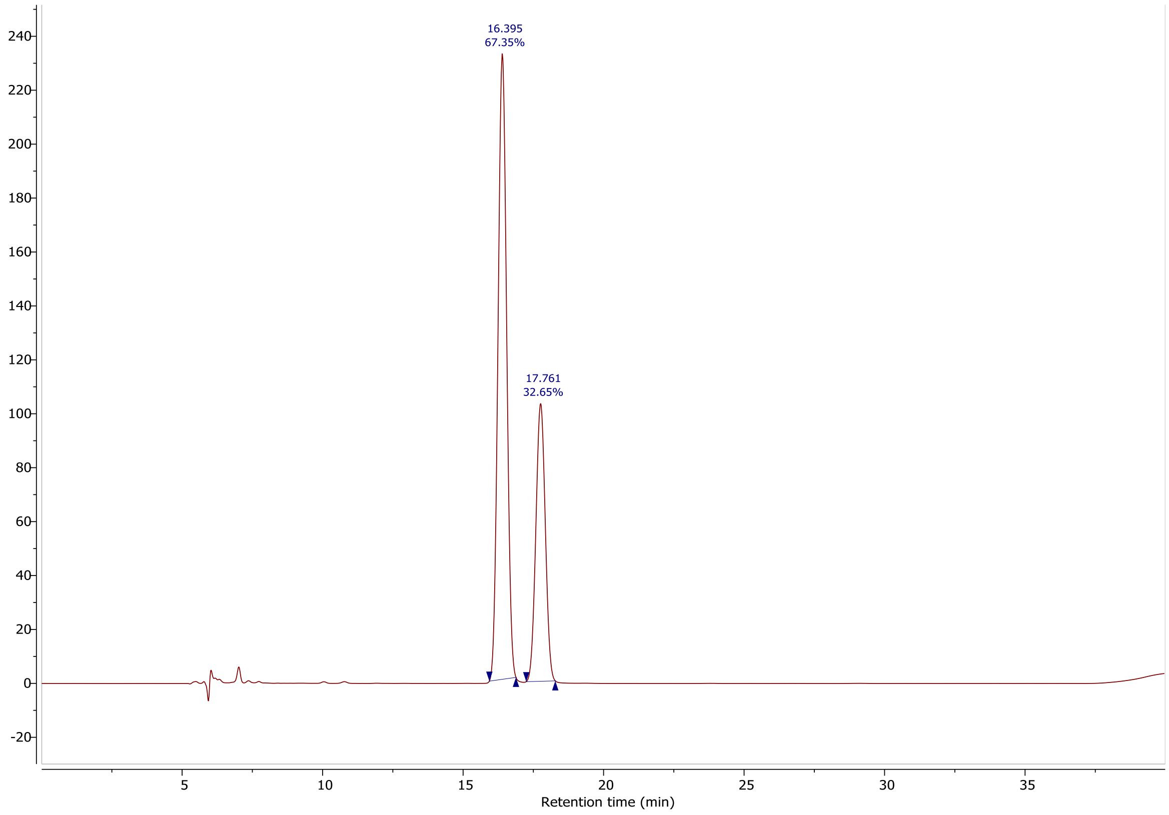

Figure S10. Chiral-LC spectrum for product **3** obtained using Sav:**1** (1 mol% + TFA).

Entry	Retention time (min)	Peak Area (%)
1 (*R*-enantiomer)	16.4	67.4
2 (*S*-enantiomer)	17.8	32.6

Sav:1 (1 mol%) at 10 °C

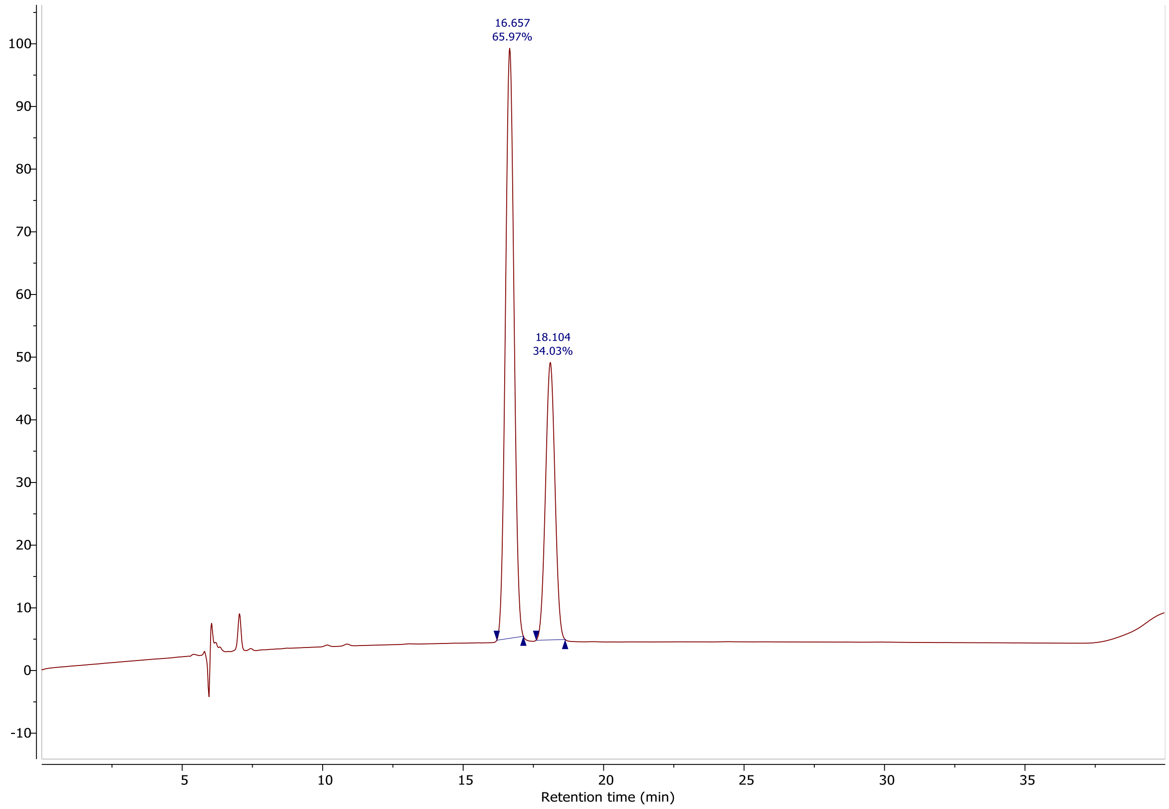

Figure S11. Chiral-LC spectrum for product **3** obtained using Sav:**1** (1 mol%, 10 °C).

Entry	Retention time (min)	Peak Area (%)
1 (*R*-enantiomer)	16.6	66.0
2 (*S*-enantiomer)	18.1	34.0

Sav:1 (1 mol%) using 5 equivalent of acetone and 25% methanol

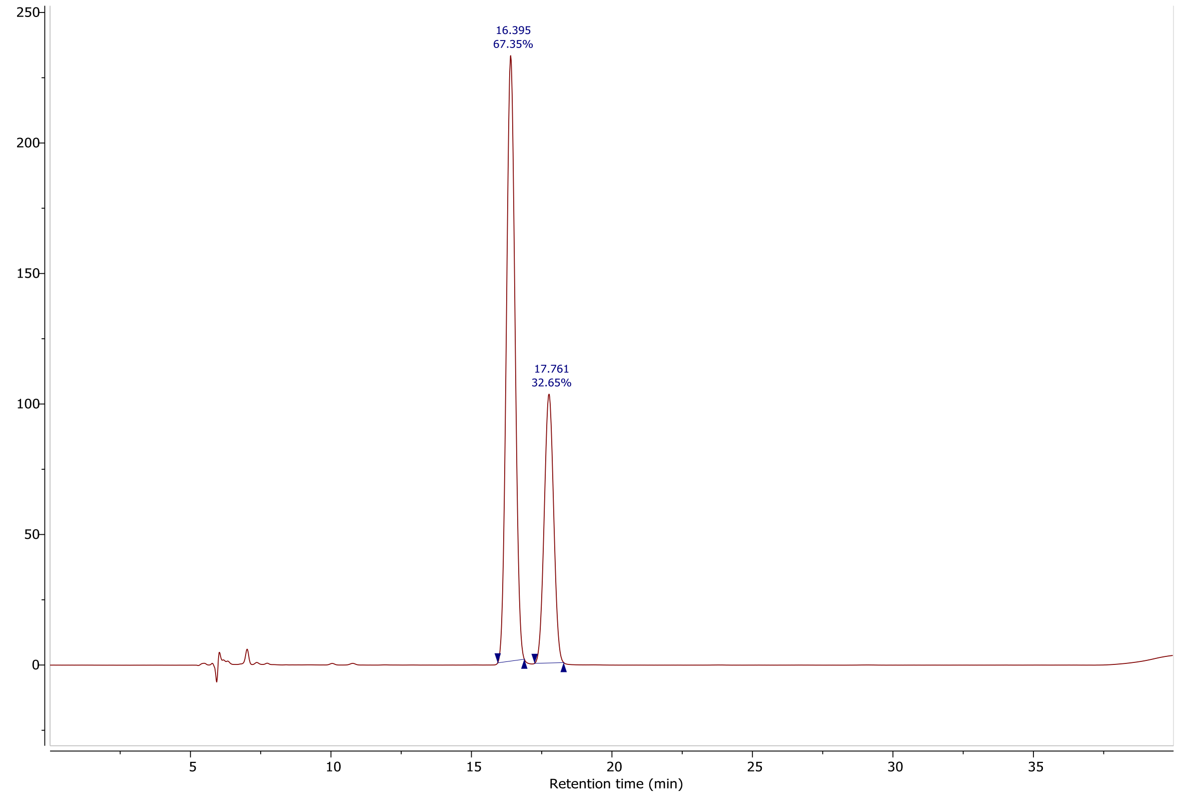

Figure S12. Chiral-LC spectrum for product **3** obtained using Sav:**1** (1 mol%, 5 equivalents of acetone, and 25% methanol).

Entry	Retention time (min)	Peak Area (%)
1 (*R*-enantiomer)	16.4	67.4
2 (*S*-enantiomer)	17.8	32.6

Sav:1 (1 mol%) using 5 equivalent of acetone and 25% acetonitrile

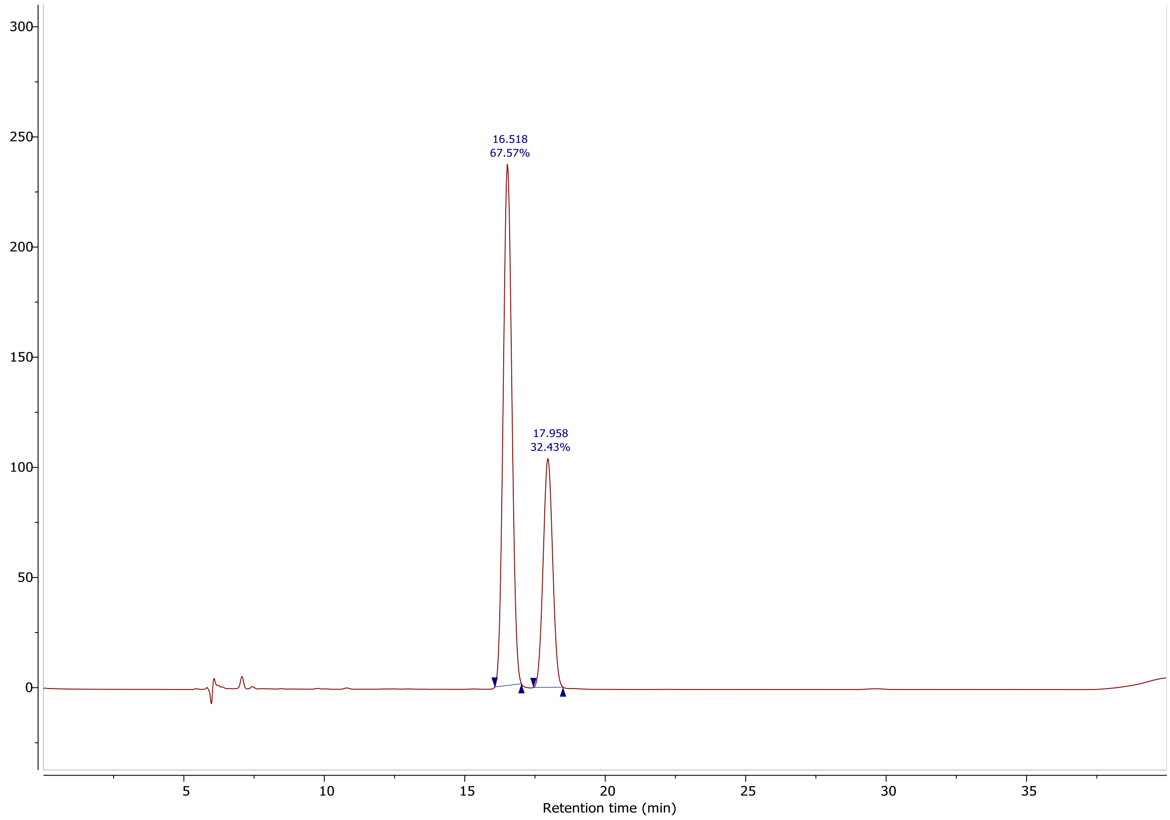

Figure S13. Chiral-LC spectrum for product **3** obtained using Sav:**1** (1 mol%, 5 equivalents of acetone, and 25% acetonitrile).

Entry	Retention time (min)	Peak Area (%)
1 (*R*-enantiomer)	16.5	67.6
2 (*S*-enantiomer)	17.9	32.4

Sav:1 (1 mol%) using 20 equivalent of acetone and 25% iso-propanol

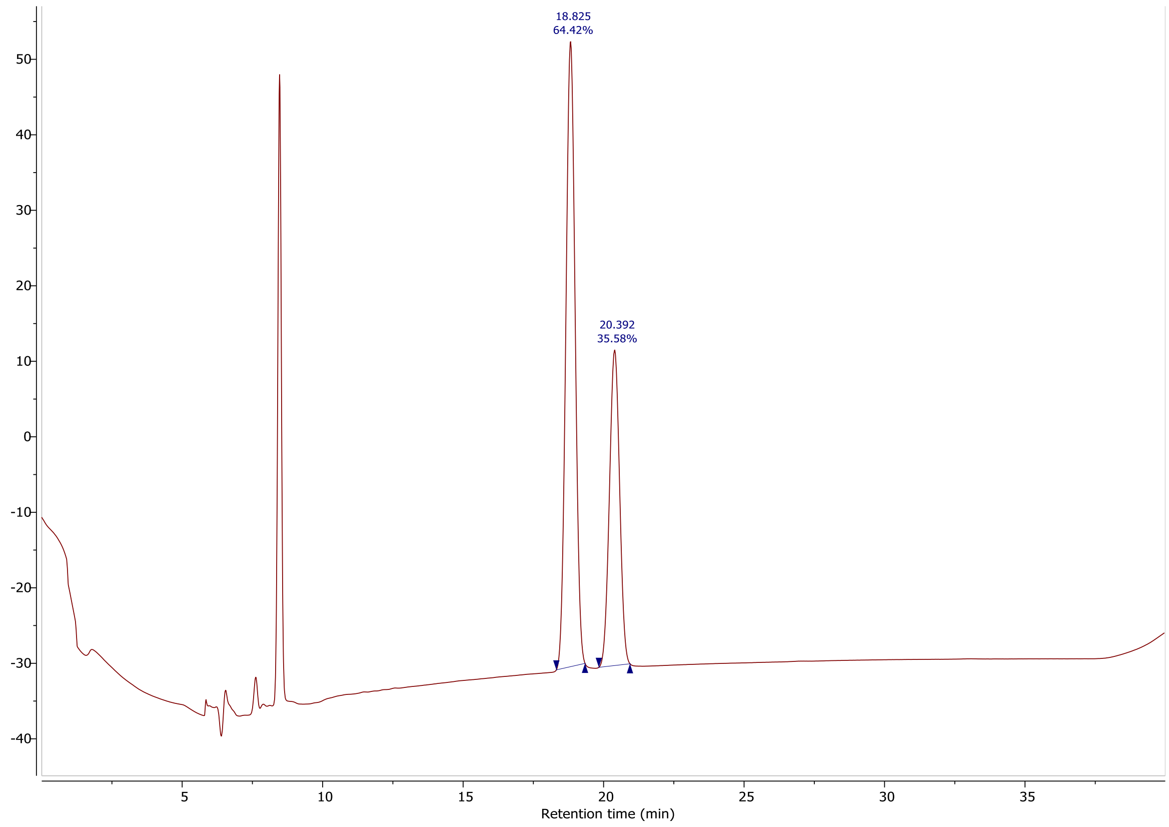

Figure S14. Chiral-LC spectrum for product **3** obtained using Sav:**1** (1 mol%, 20 equivalents of acetone, and 25% iso-propanol).

Entry	Retention time (min)	Peak Area (%)
1 (*R*-enantiomer)	18.8	64.4
2 (*S*-enantiomer)	20.4	35.6

Sav:1 (1 mol%) using 50 equivalent of acetone and 25% iso-propanol

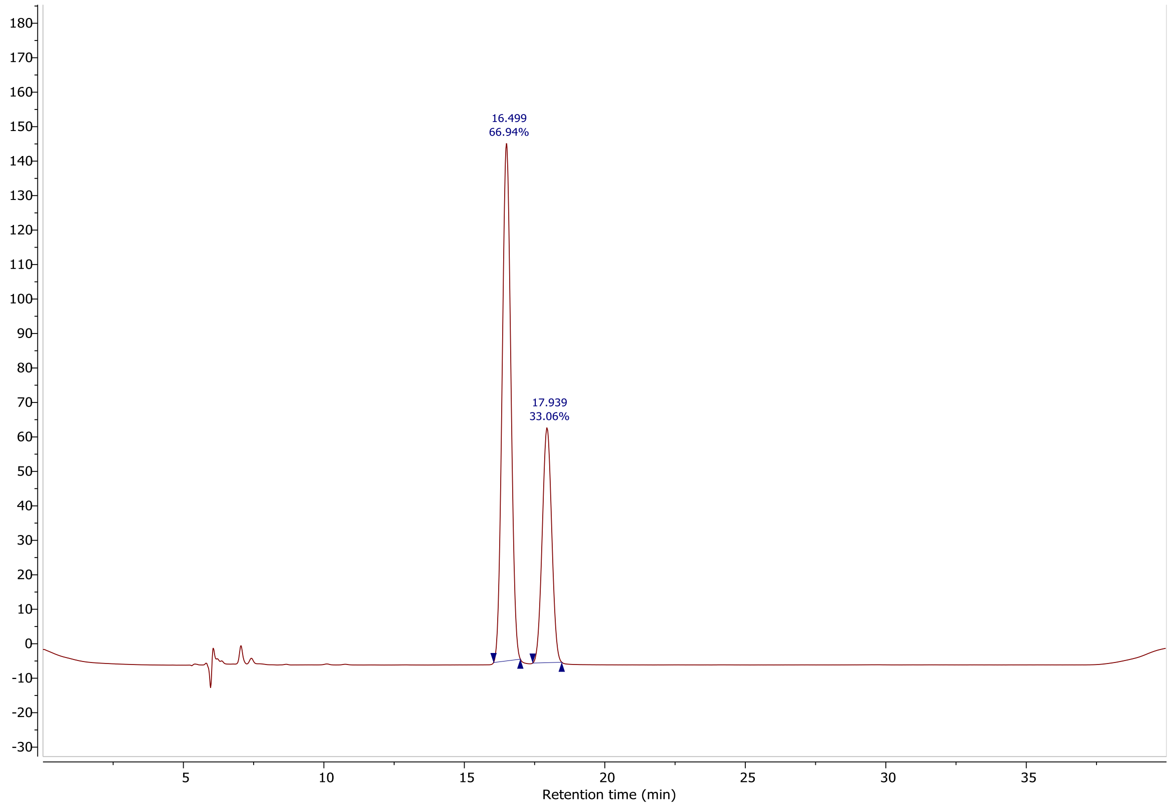

Figure S15. Chiral-LC spectrum for product **3** obtained using Sav:**1** (1 mol%, 50 equivalents of acetone, and 25% methanol).

Entry	Retention time (min)	Peak Area (%)
1 (*R*-enantiomer)	16.4	66.9
2 (*S*-enantiomer)	17.9	33.1

T-rSav and mutants:1 Reactions

T-rSav:1

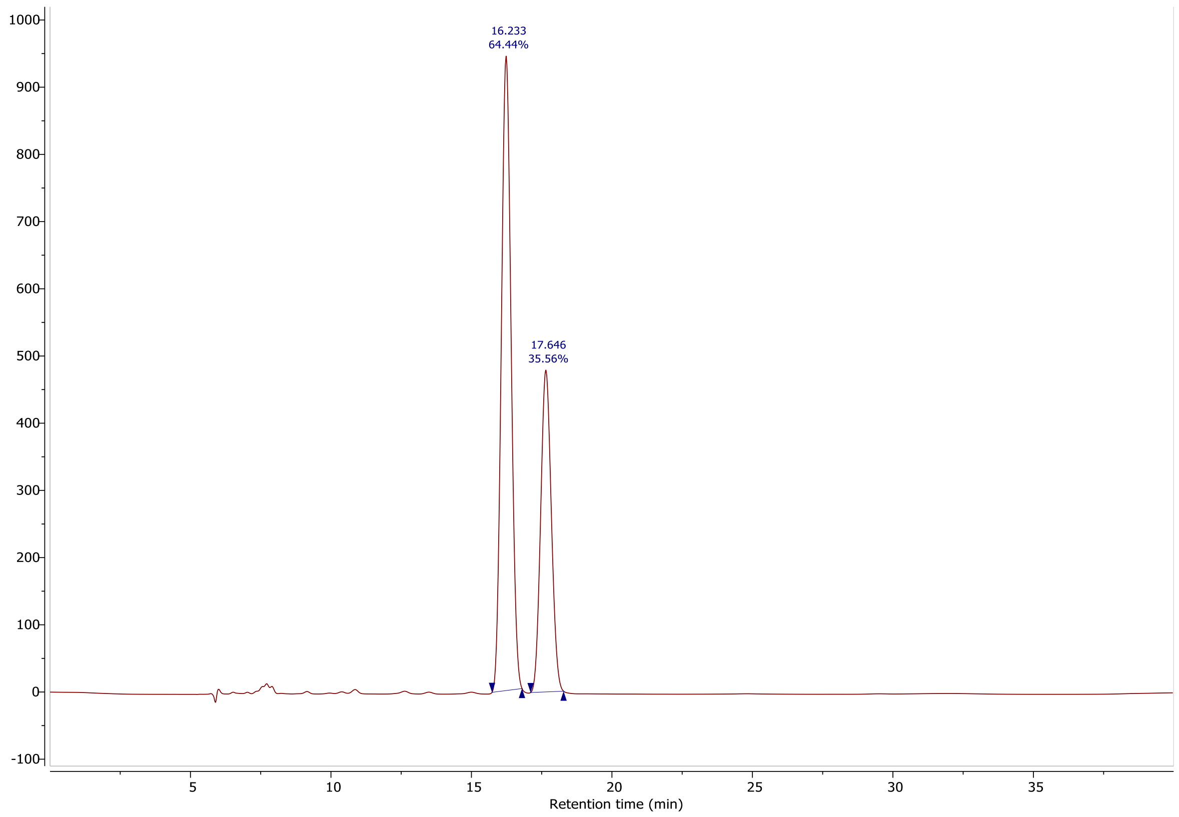

Figure S16. Chiral-LC spectrum for product **3** obtained using T-rSav:**1** (1 mol%).

Entry	Retention time (min)	Peak Area (%)
1 (*R*-enantiomer)	16.2	64.4
2 (*S*-enantiomer)	17.6	35.6

S112E:1

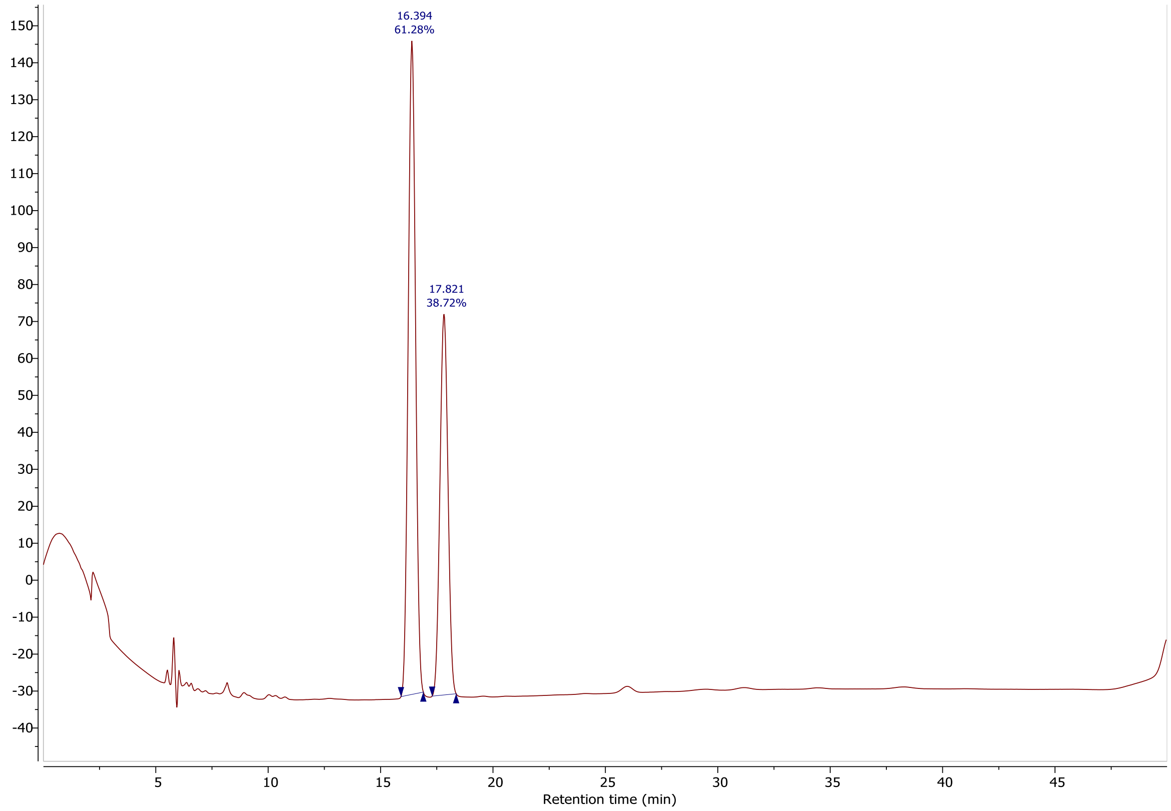

Figure S17. Chiral-LC spectrum for product **3** obtained using S112E:**1** (1 mol%).

Entry	Retention time (min)	Peak Area (%)
1 (*R*-enantiomer)	16.4	61.3
2 (*S*-enantiomer)	17.8	38.7

K121A:1

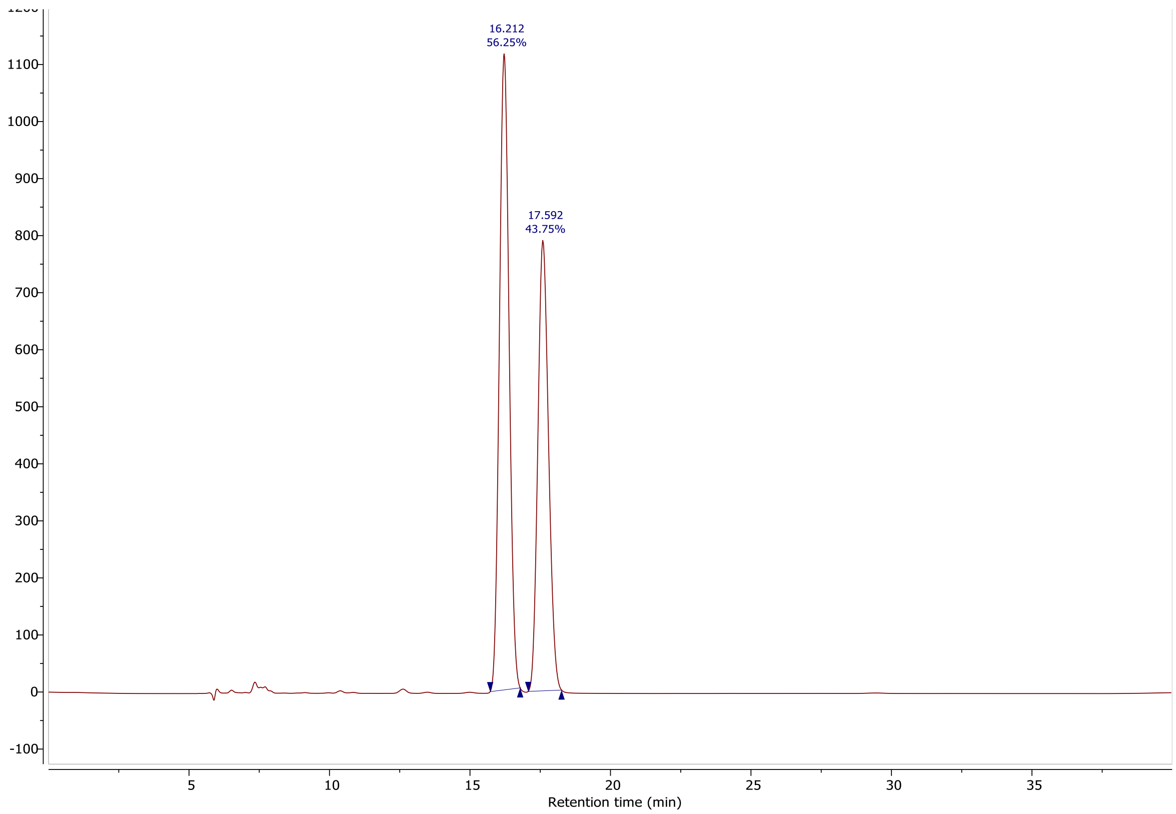

Figure S18. Chiral-LC spectrum for product **3** obtained using K121A:**1** (1 mol%).

Entry	Retention time (min)	Peak Area (%)
1 (*R*-enantiomer)	16.2	56.2
2 (*S*-enantiomer)	17.6	43.8

^{1}H NMR Details for the Activity Screening of Catalysts 1 for the Aldol Addition Reaction of Acetone and *p*-Nitrobenzaldehyde

Screening Reactions

^{1}H NMR Spectrum after Extraction of the Reaction Mixture between *p*-Nitrobenzaldehyde and Acetone (no catalyst)

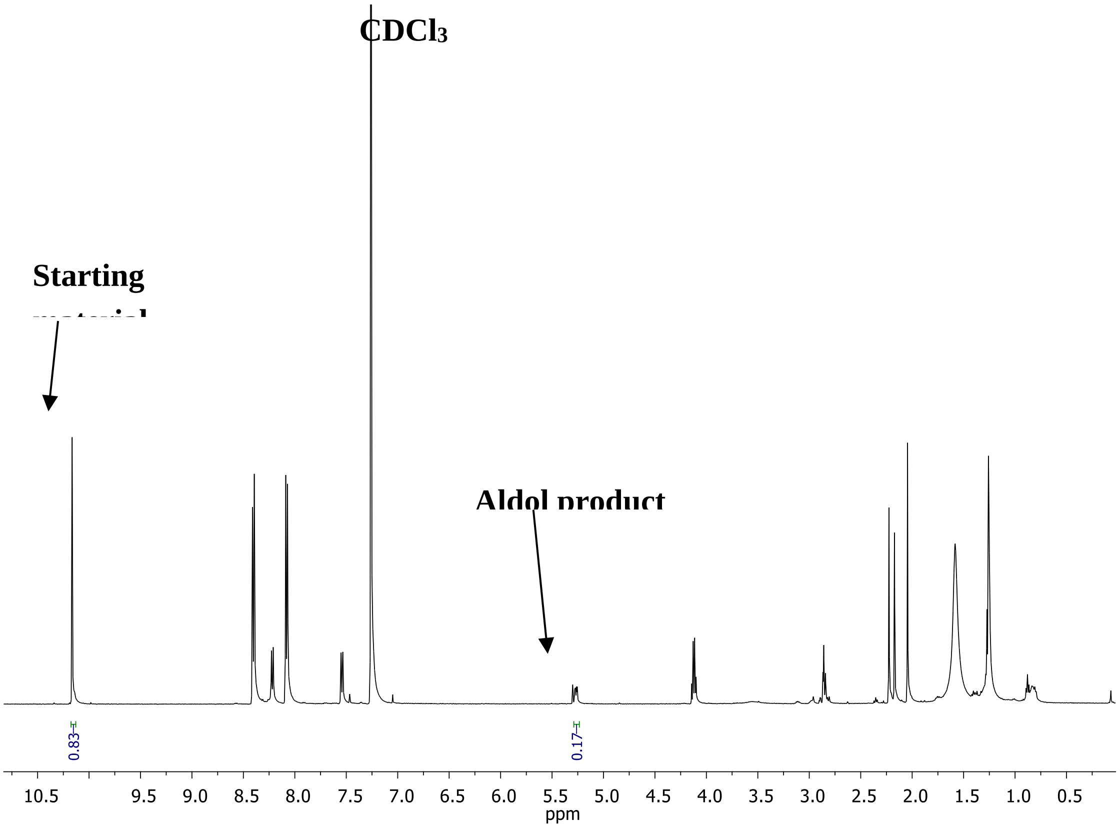

Figure S19. ^{1}H NMR spectrum for the crude of reaction using no catalyst.

^{1}H NMR Spectrum after Extraction of the Reaction Mixture between *p*-Nitrobenzaldehyde and Acetone (catalyst 1)

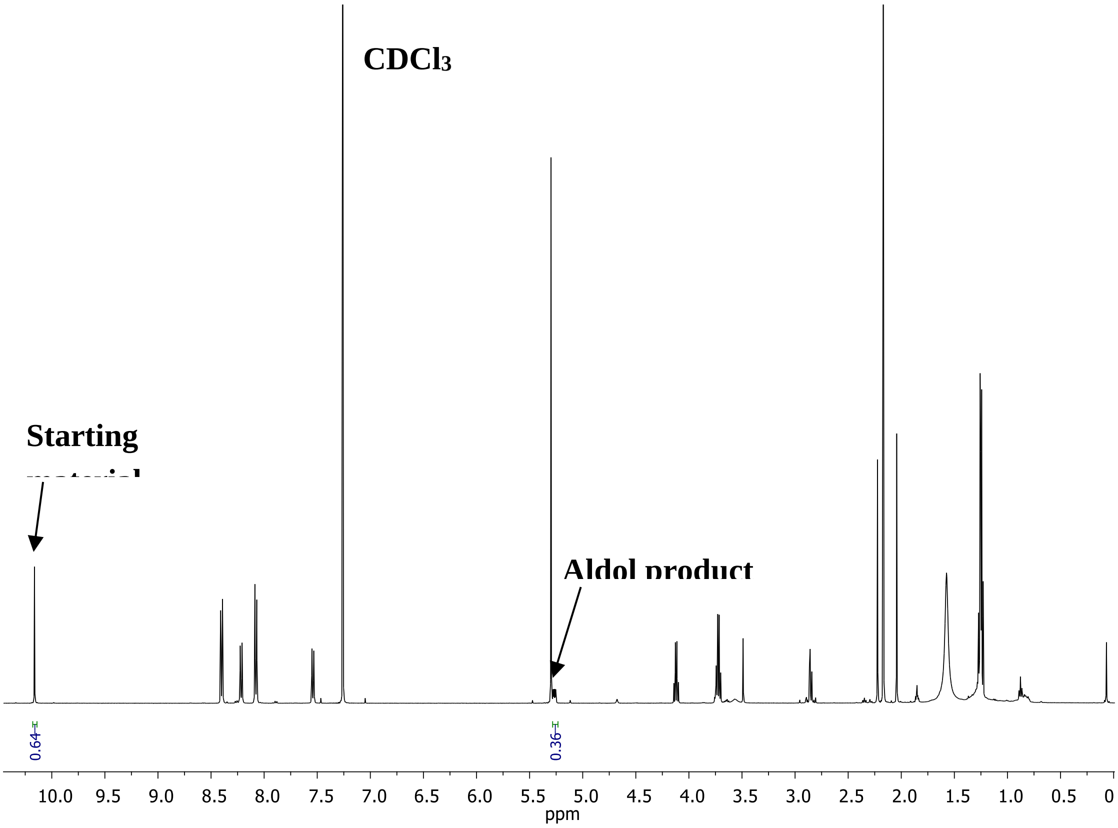

Figure S20. ^{1}H NMR spectrum for the crude of reaction using catalyst **1**.

^{1}H NMR Spectrum after Extraction of the Reaction Mixture between *p*-Nitrobenzaldehyde and Acetone (Sav, 0.1 mol%)

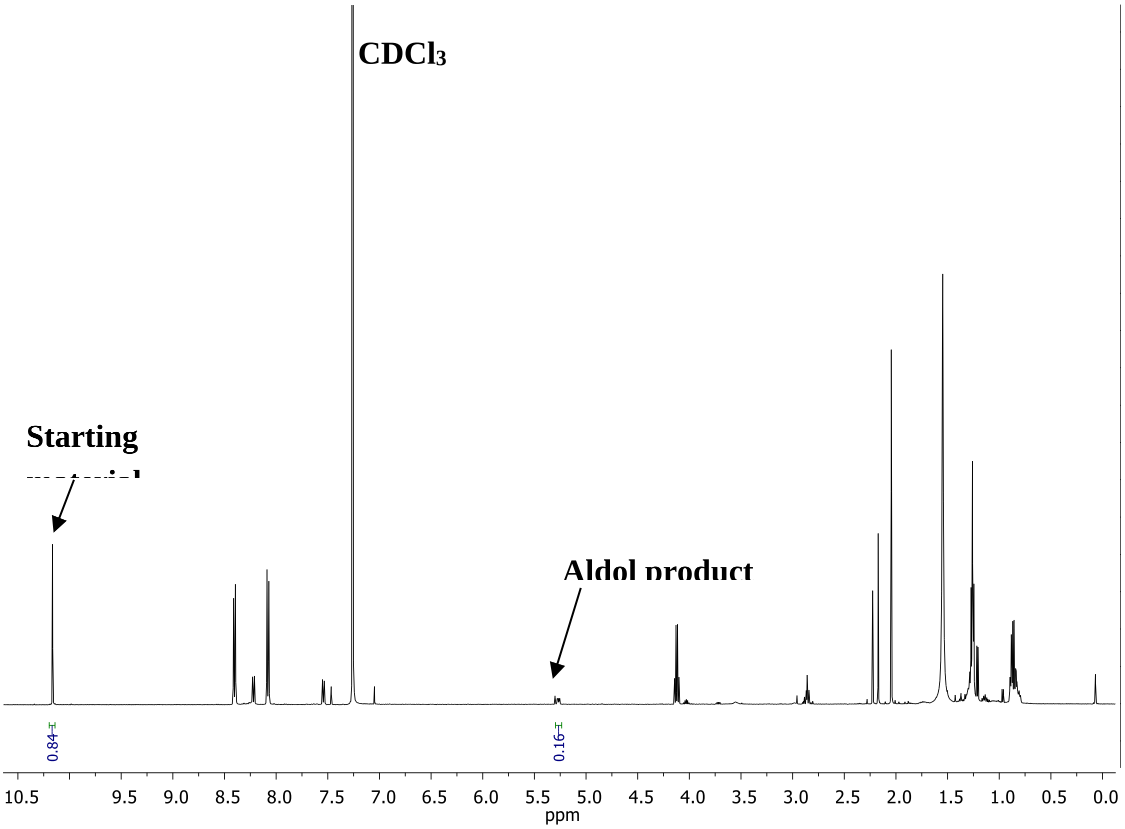

Figure S21. ^{1}H NMR spectrum for the crude of reaction using Sav (0.1 mol%).

^{1}H NMR Spectrum after Extraction of the Reaction Mixture between *p*-Nitrobenzaldehyde and Acetone (Sav, 0.5 mol%)

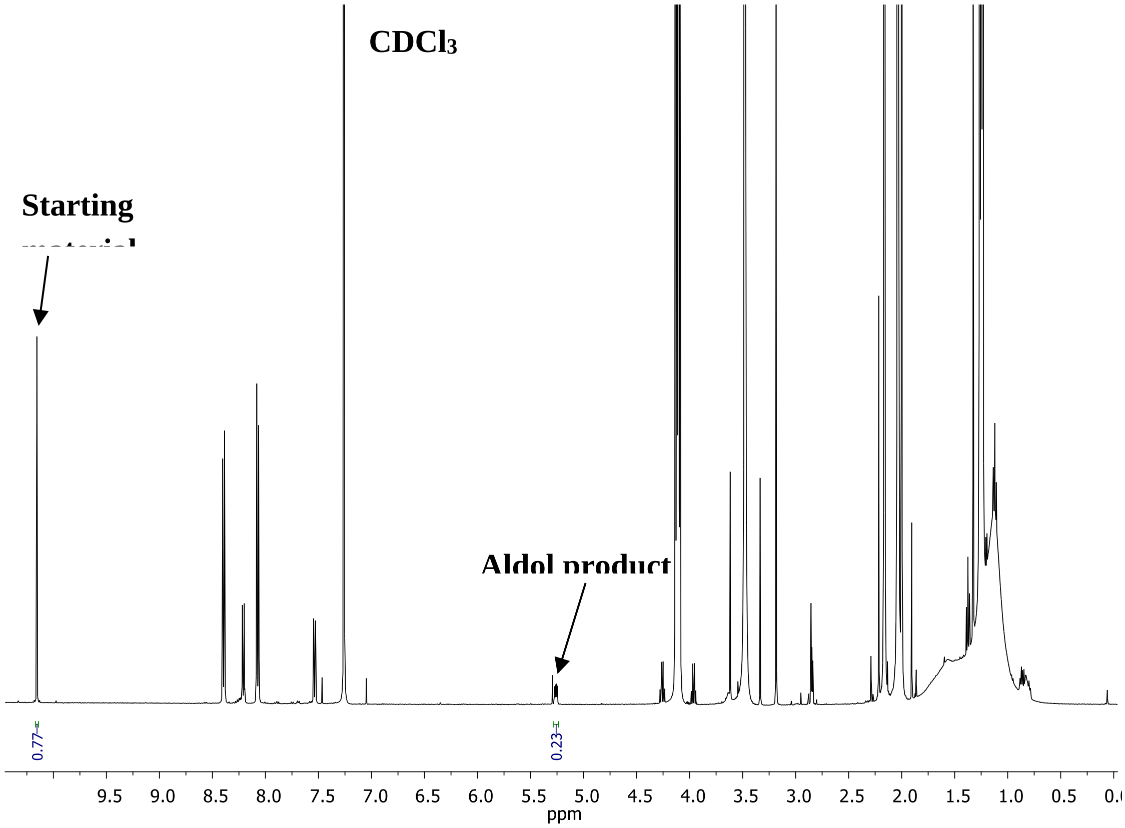

Figure S22. ^{1}H NMR spectrum for the crude of reaction using Sav (0.5 mol%).

^{1}H NMR Spectrum after Extraction of the Reaction Mixture between *p*-Nitrobenzaldehyde and Acetone (Sav, 1 mol%)

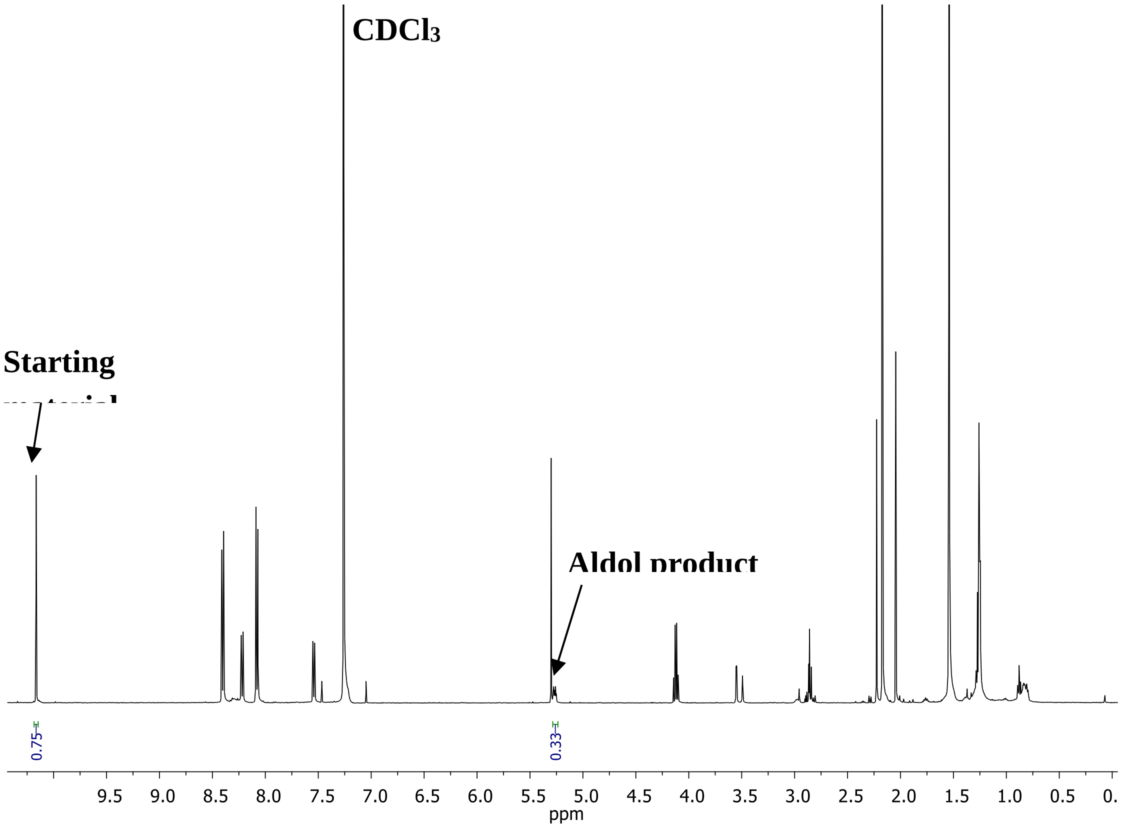

Figure S23. ^{1}H NMR spectrum for the crude of reaction using Sav (1 mol%).

^{1}H NMR Spectrum after Extraction of the Reaction Mixture between *p*-Nitrobenzaldehyde and Acetone (Sav:1, 0.1 mol%)

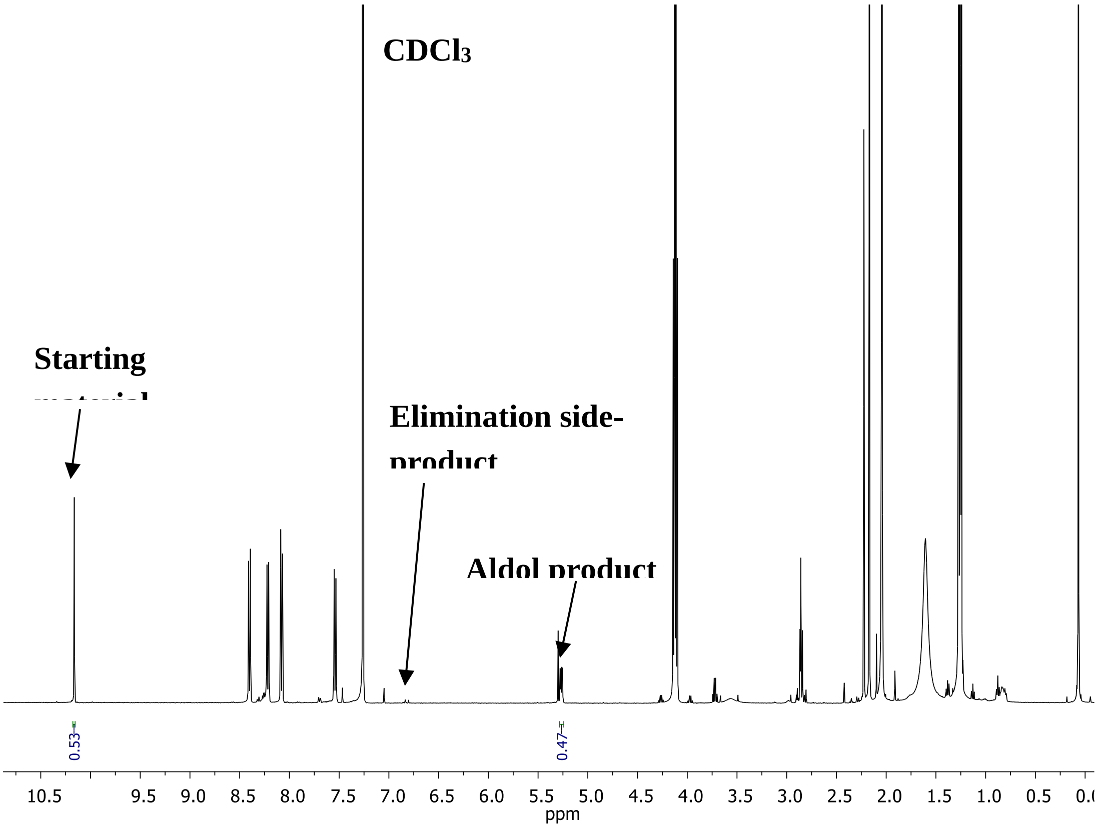

Figure S24. ^{1}H NMR spectrum for the crude of reaction using Sav:**1** (0.1 mol%).

^{1}H NMR Spectrum after Extraction of the Reaction Mixture between *p*-Nitrobenzaldehyde and Acetone (Sav:1, 0.5 mol%)

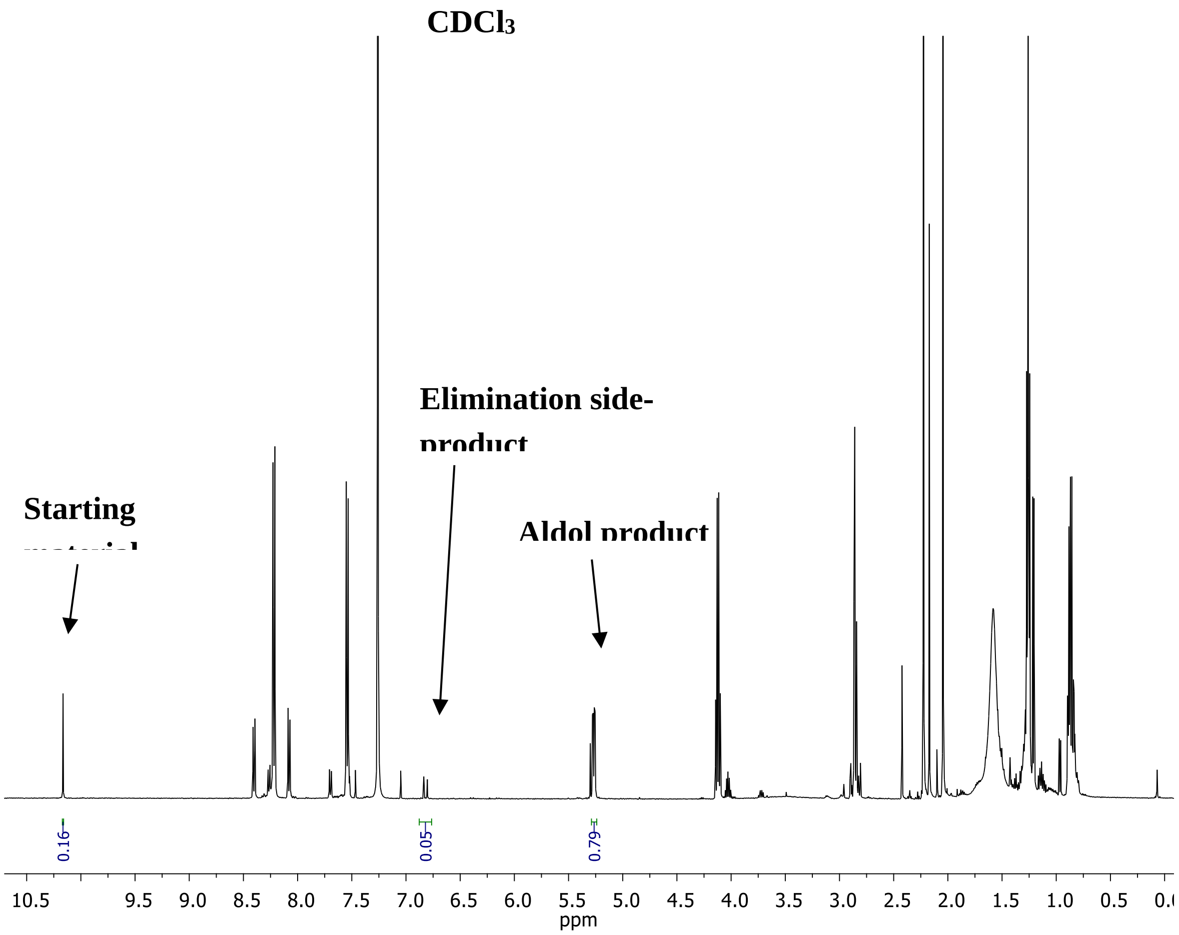

Figure S25. ^{1}H NMR spectrum for the crude of reaction using Sav:**1** (0.5 mol%).

^{1}H NMR Spectrum after Extraction of the Reaction Mixture between *p*-Nitrobenzaldehyde and Acetone (Sav:1, 1 mol%)

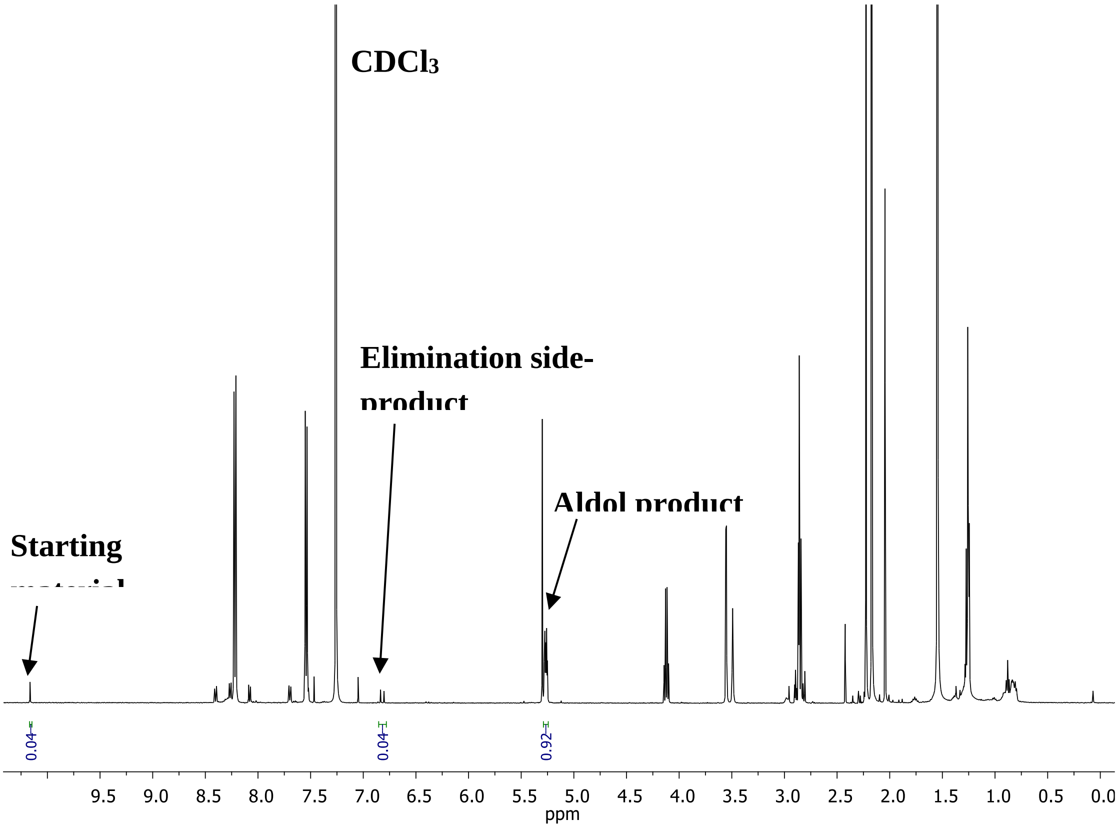

Figure S26. ^{1}H NMR spectrum for the crude of reaction using Sav:**1** (1 mol%).

^{1}H NMR Spectrum after Extraction of the Reaction Mixture between *p*-Nitrobenzaldehyde and Acetone (Sav:1, 1 mol%, and TFA 1 mol%)

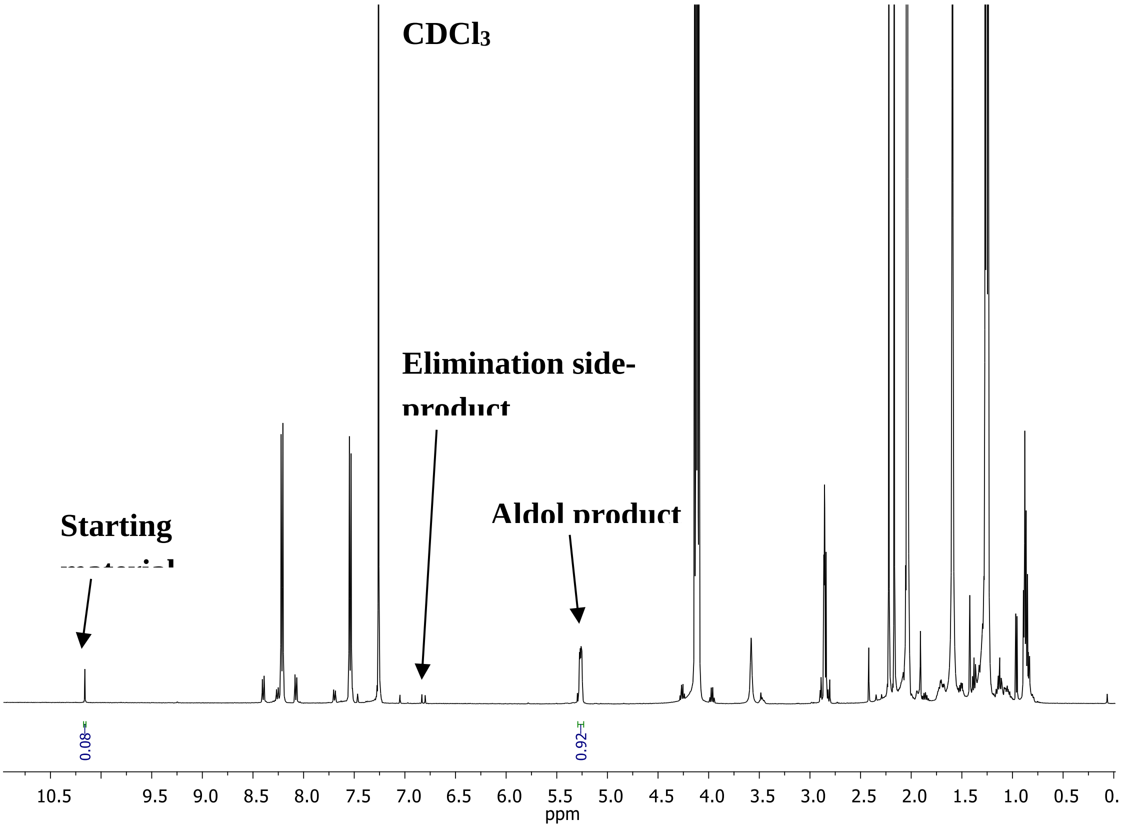

Figure S27. ^{1}H NMR spectrum for the crude of reaction using Sav:**1** (1 mol% + TFA).

^{1}H NMR Spectrum after Extraction of the Reaction Mixture between *p*-Nitrobenzaldehyde and Acetone (Sav:1, 1 mol%) at 10 °C

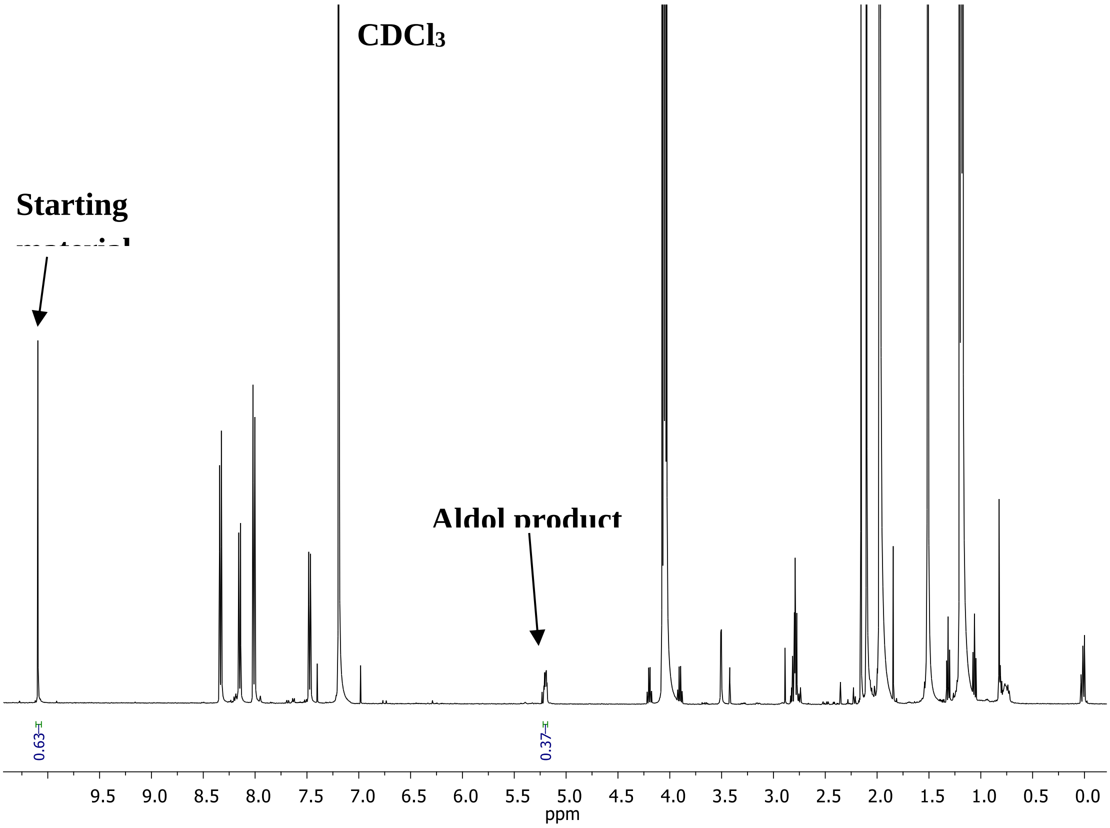

Figure S28. ^{1}H NMR spectrum for the crude of reaction using Sav:**1** (1 mol%, 10 °C).

^{1}H NMR Spectrum after Extraction of the Reaction Mixture between *p*-Nitrobenzaldehyde and 5 Equivalents of Acetone (Sav:1, 1 mol%, 25% Methanol)

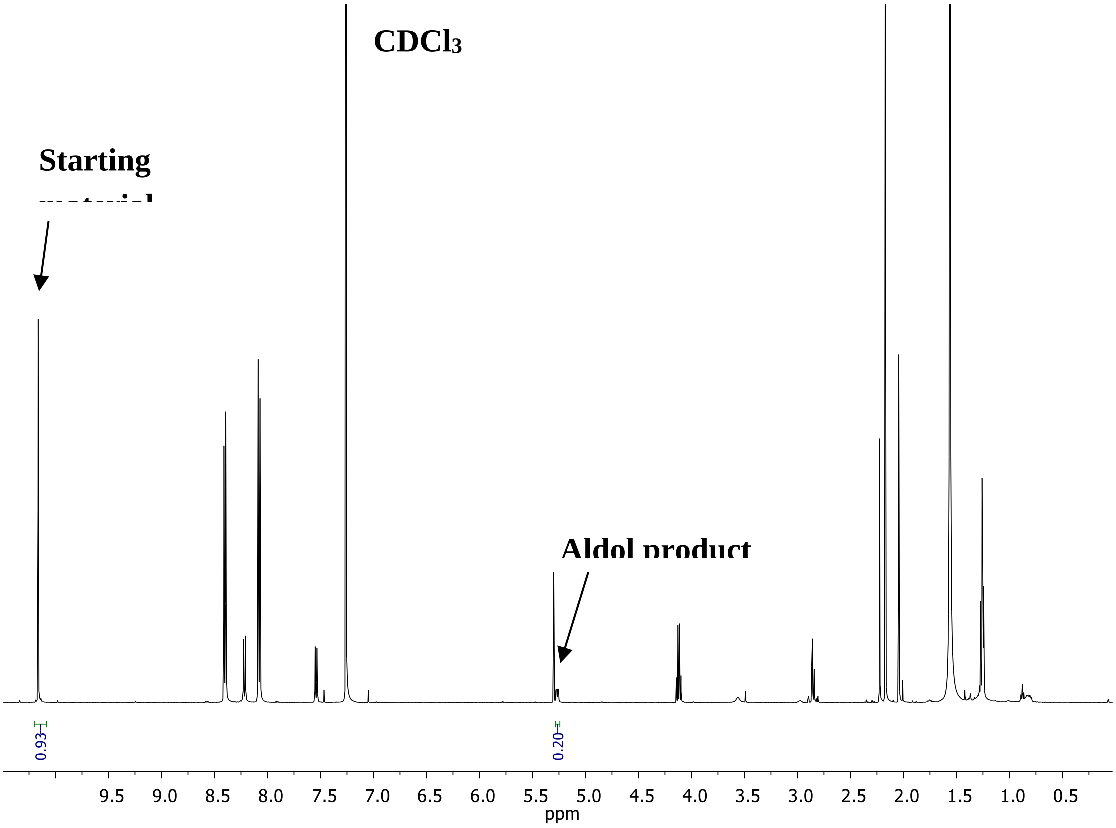

Figure S29. ^{1}H NMR spectrum for the crude of reaction using Sav:**1** (1 mol%, 5 equivalents of acetone and 25% methanol).

^{1}H NMR Spectrum after Extraction of the Reaction Mixture between *p*-Nitrobenzaldehyde and 5 Equivalents of Acetone (Sav:1, 1 mol%, 25% Acetonitrile)

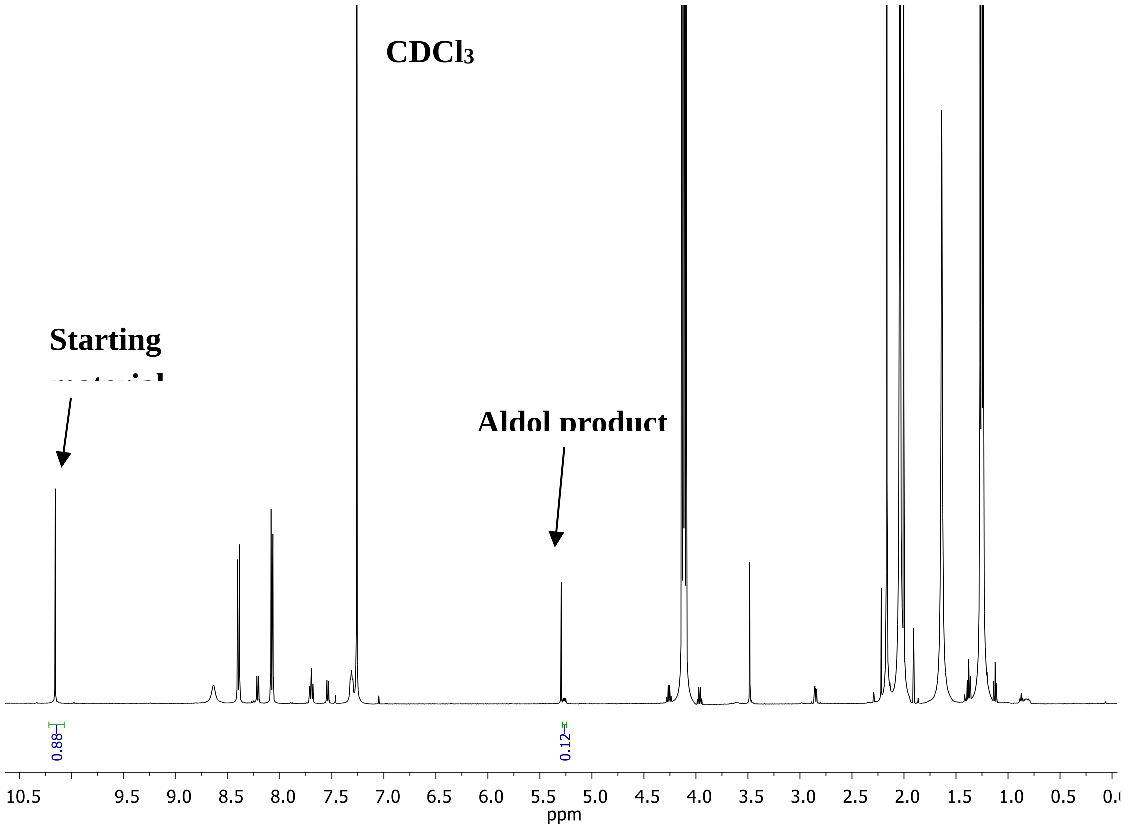

Figure S30. ^{1}H NMR spectrum for the crude of reaction using Sav:**1** (1 mol%, 5 equivalents of acetone and 25% acetonitrile).

^{1}H NMR Spectrum after Extraction of the Reaction Mixture between *p*-Nitrobenzaldehyde and 5 Equivalents of Acetone (Sav:1, 1 mol%, 25% Iso-Propanol)

Integrations of starting material and product has been made using the aromatic peaks as standard.

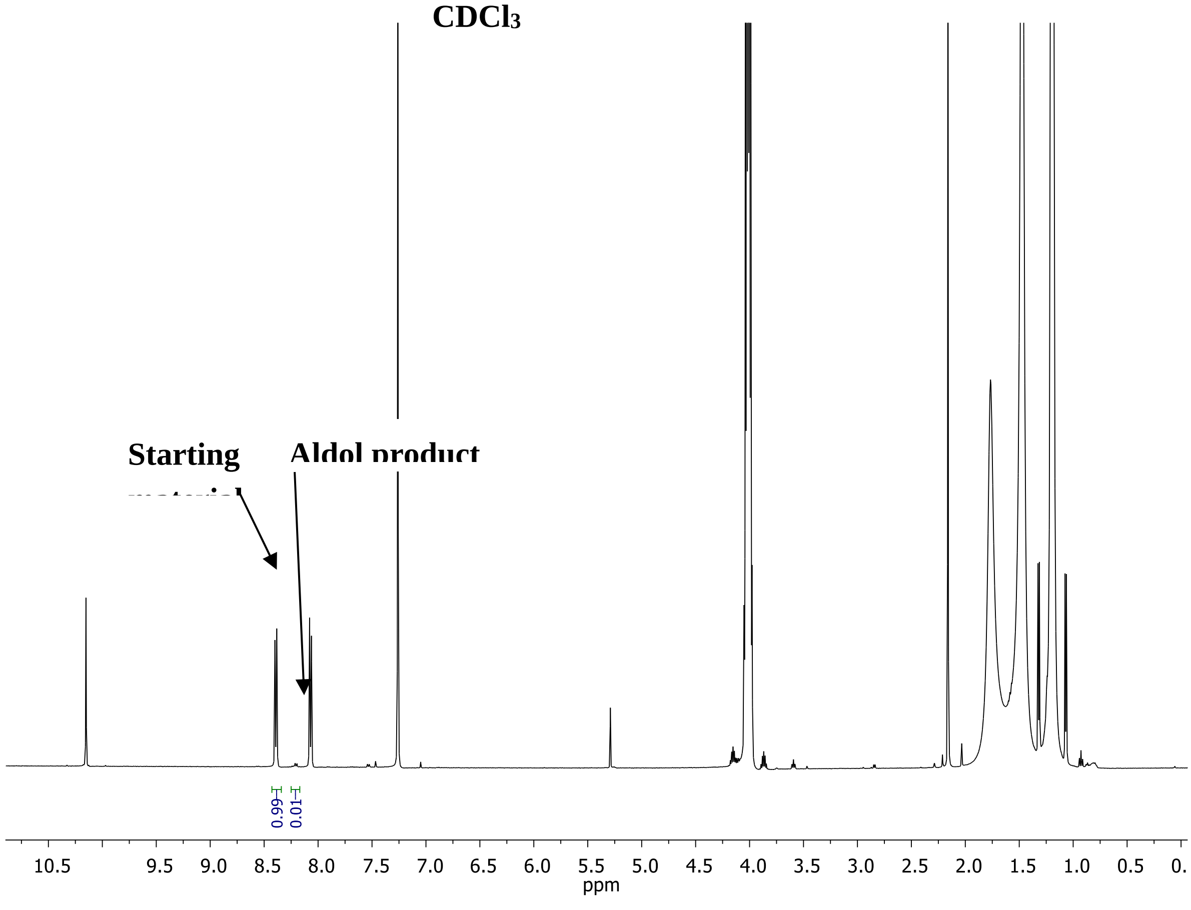

Figure S31. ^{1}H NMR spectrum for the crude of reaction using Sav:**1** (1 mol%, 5 equivalents of acetone and 25% iso-propanol).

^{1}H NMR Spectrum after Extraction of the Reaction Mixture between *p*-Nitrobenzaldehyde and 10 Equivalents of Acetone (Sav:1, 1 mol%, 25% Iso-Propanol)

Integrations of starting material and product has been made using the aromatic peaks as standard.

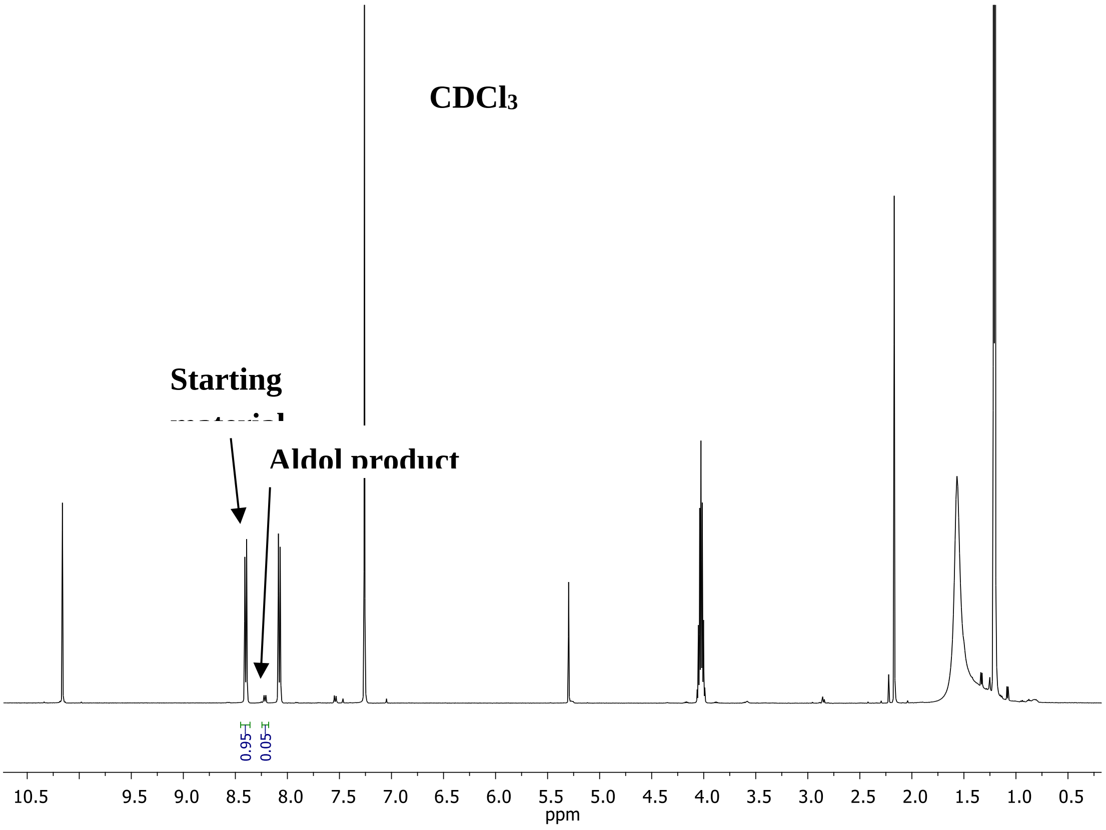

Figure S32. ^{1}H NMR spectrum for the crude of reaction using Sav:**1** (1 mol%, 10 equivalents of acetone and 25% iso-propanol).

¹H NMR Spectrum after Extraction of the Reaction Mixture between *p*-Nitrobenzaldehyde and 20 Equivalents of Acetone (Sav:1, 1 mol%, 25% Iso-Propanol)

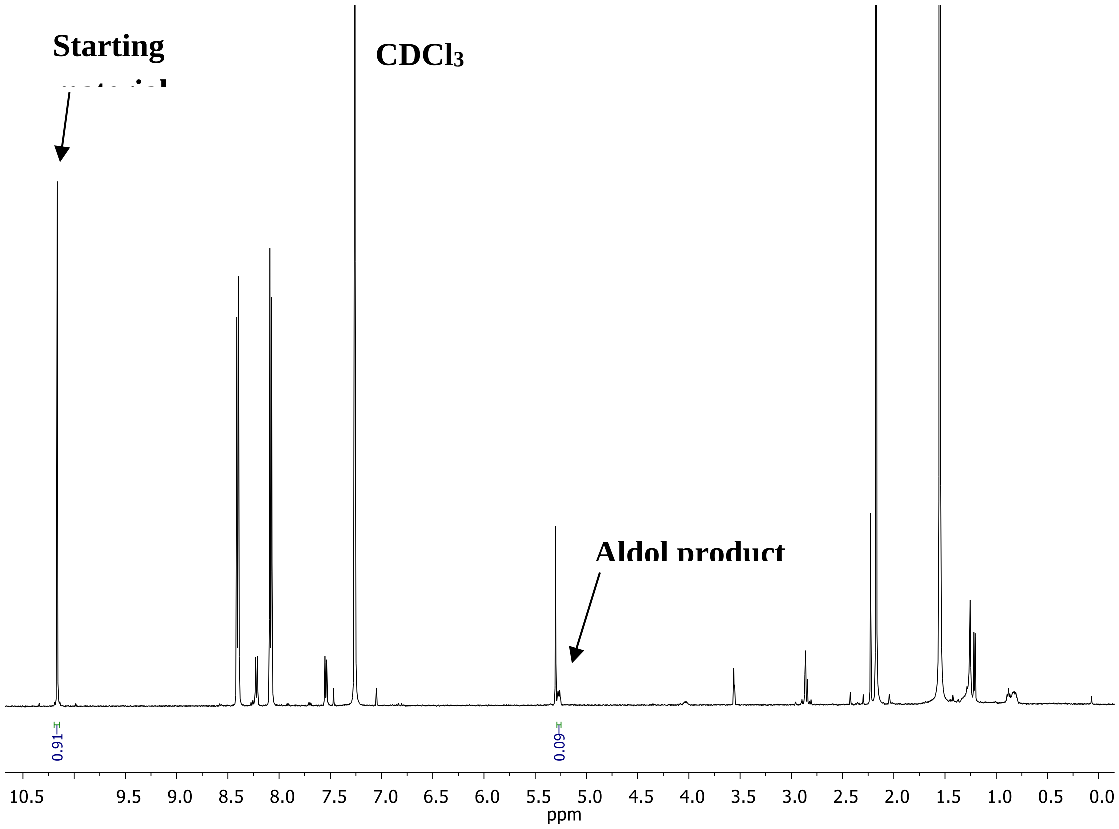

Figure S33. ¹H NMR spectrum for the crude of reaction using Sav:**1** (1 mol%, 20 equivalents of acetone and 25% iso-propanol).

^{1}H NMR Spectrum after Extraction of the Reaction Mixture between *p*-Nitrobenzaldehyde and 50 Equivalents of Acetone (Sav:1, 1 mol%, 25% Iso-Propanol)

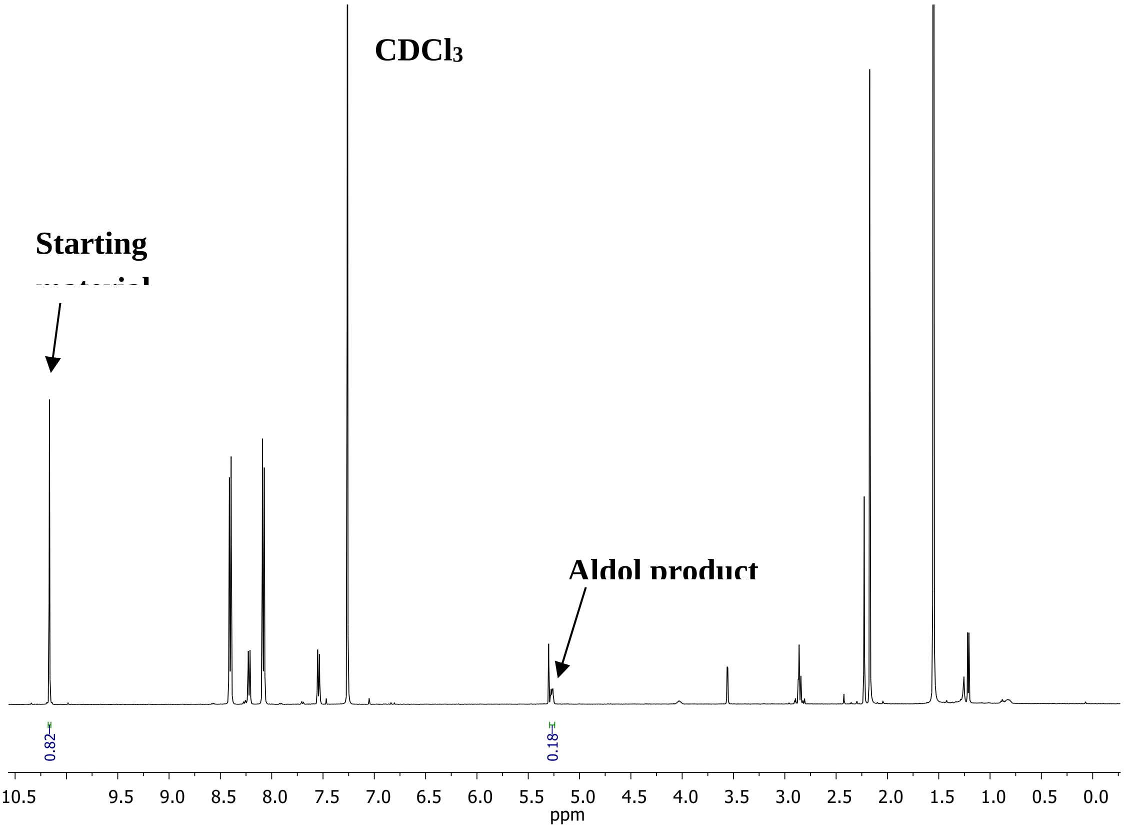

Figure S34. ^{1}H NMR spectrum for the crude of reaction using Sav:**1** (1 mol%, 50 equivalents of acetone and 25% iso-propanol).

Protein scope

^{1}H NMR Spectrum after Extraction of the Reaction Mixture between *p*-Nitrobenzaldehyde and Acetone (T-rSav:1, 1 mol%)

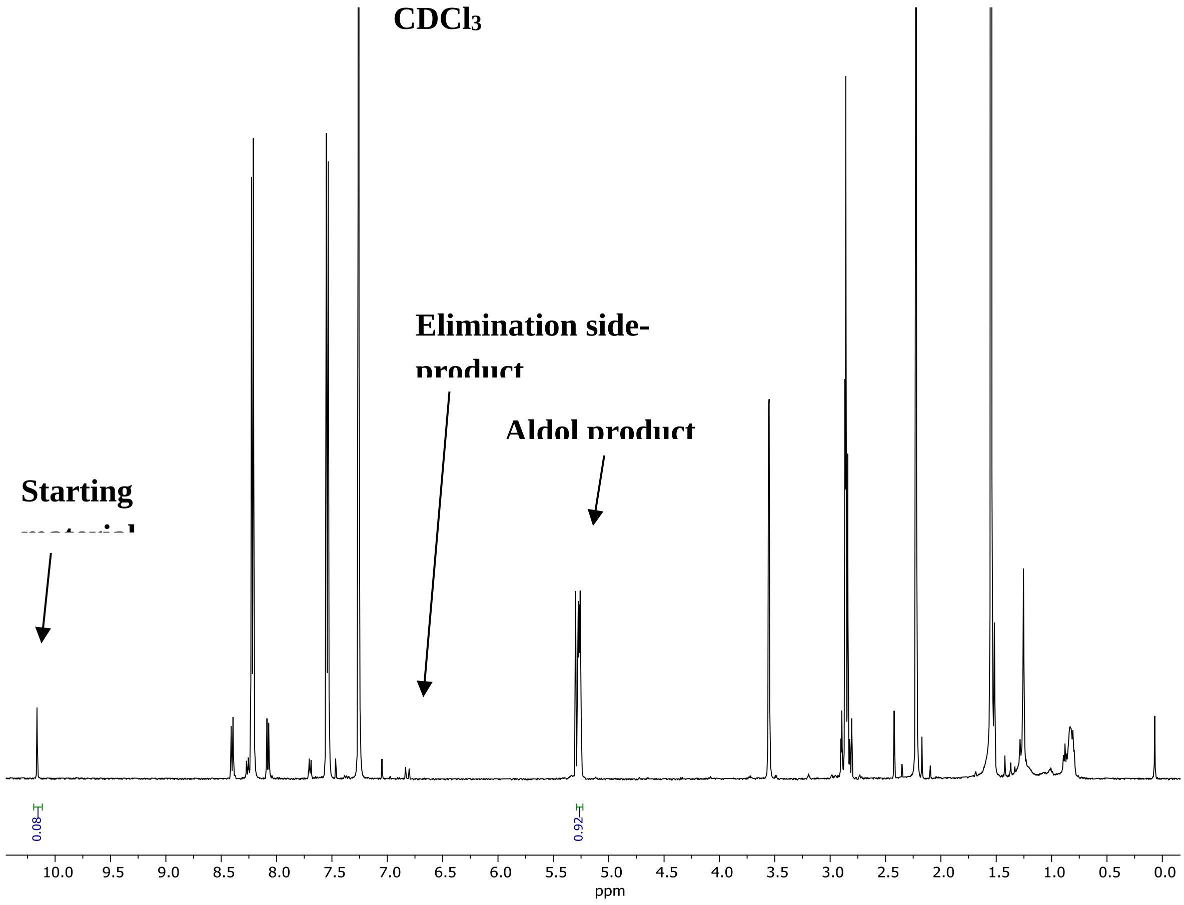

Figure S35. ^{1}H NMR spectrum for the crude of reaction using T-rSav:**1** (1 mol%).

^{1}H NMR Spectrum after Extraction of the Reaction Mixture between *p*-Nitrobenzaldehyde and Acetone (S112E:1, 1 mol%)

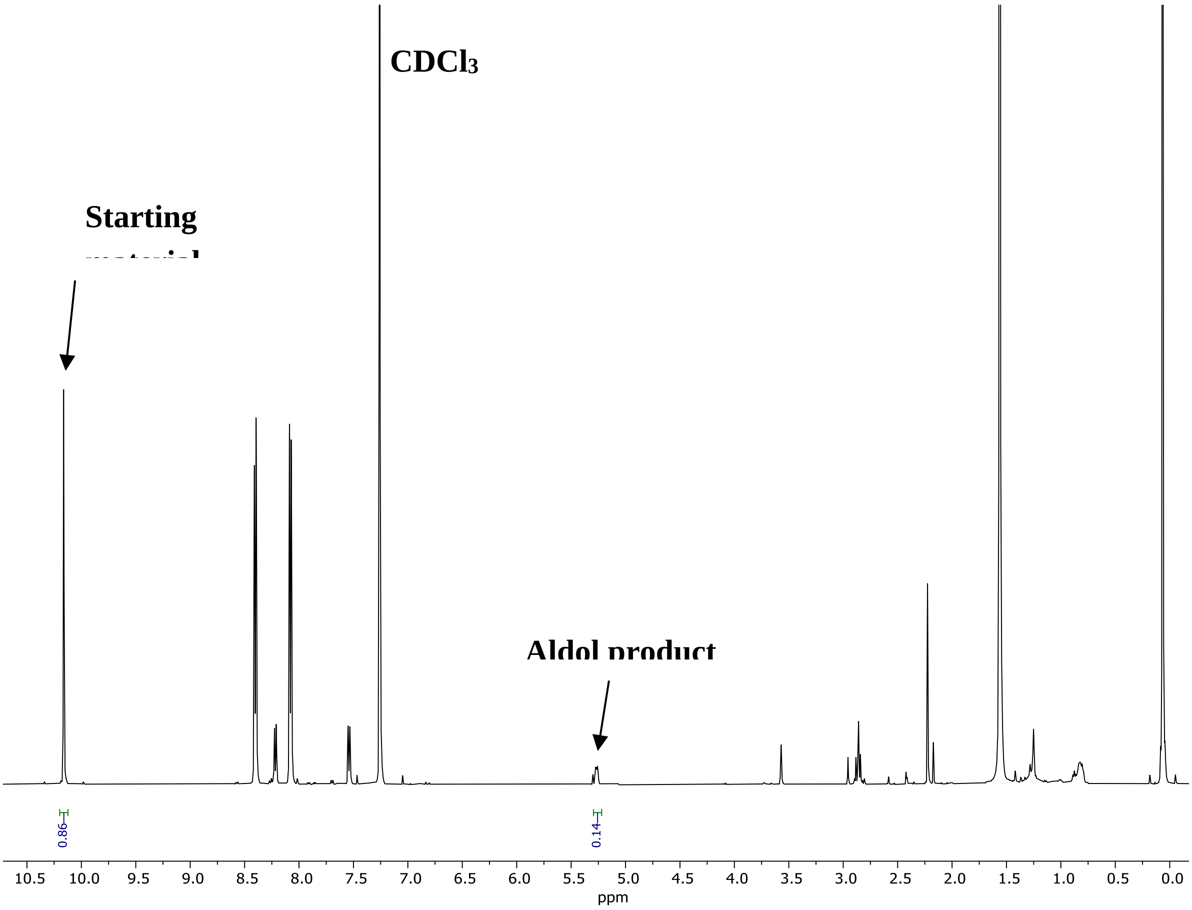

Figure S36. ^{1}H NMR spectrum for the crude of reaction using S112E:**1** (1 mol%).

^{1}H NMR Spectrum after Extraction of the Reaction Mixture between *p*-Nitrobenzaldehyde and Acetone (K121A:1, 1 mol%)

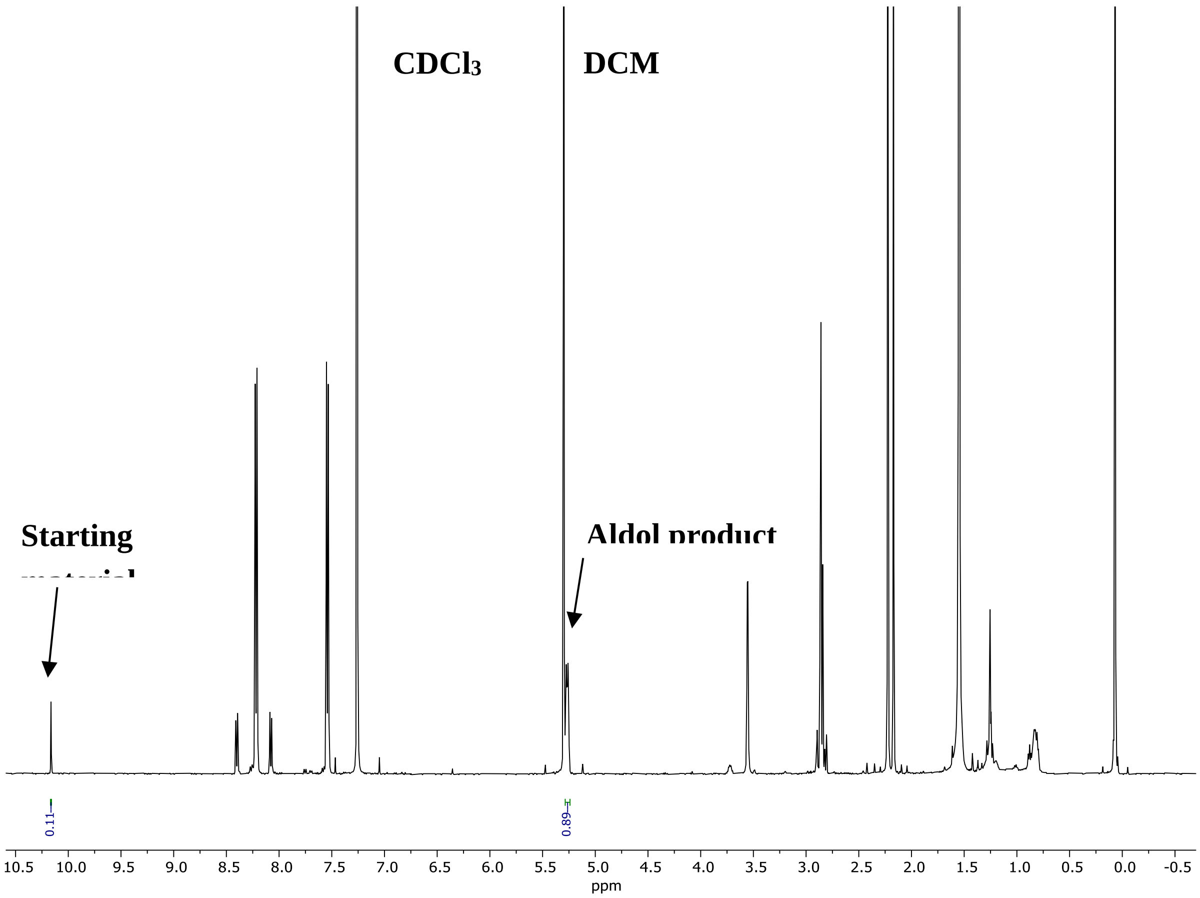

Figure S37. ^{1}H NMR spectrum for the crude of reaction using K121A:**1** (1 mol%).
